# Micellar Solvent Accessibility of Esterified Polyoxyethylene Chains as Crucial Element of Polysorbate Oxidation: A Density Functional Theory, Molecular Dynamics Simulation and Liquid Chromatography/Mass Spectrometry Investigation

**DOI:** 10.1021/acs.molpharmaceut.4c01015

**Published:** 2025-02-03

**Authors:** Johanna Weber, Leonardo Pedri, Luis P. Peters, Patrick K. Quoika, Dennis F. Dinu, Klaus R. Liedl, Christofer S. Tautermann, Tim Diederichs, Patrick Garidel

**Affiliations:** † Department of General, Inorganic and Theoretical Chemistry, University of Innsbruck, Innrain 80, Innsbruck 6020, Austria; ‡ Institute of Pharmacy, Faculty of Biosciences, Martin-Luther-University Halle-Wittenberg, Wolfgang-Langenbeck-Strasse 4, Halle 06120, Germany; § Center for Protein Assemblies (CPA), Physics Department, Chair of Theoretical Biophysics, Technical University of Munich, Garching 85748, Germany; ∥ Boehringer Ingelheim Pharma GmbH & Co.KG, Innovation Unit, PDB-TIP, Biberach/Riss 88400, Germany; ⊥ Medicinal Chemistry, Boehringer Ingelheim Pharma GmbH & Co. KG, Birkendorfer Straße 65, Biberach/Riss 88400, Germany

**Keywords:** biotherapeutic formulations, cDFT, MD, polysorbate micelles, tween, oxidation, protein stabilization

## Abstract

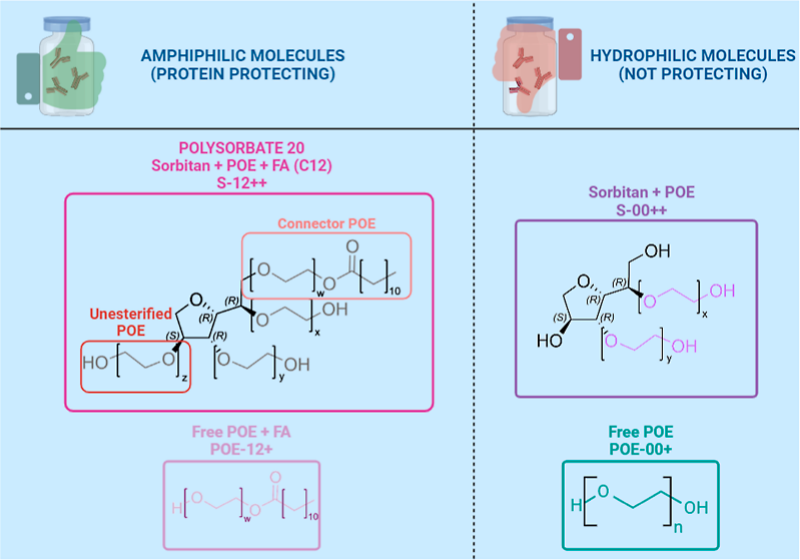

Given that the amphiphilicity of polysorbates represents a key factor in the protection of proteins from particle formation, the loss of this property through degradative processes is a significant concern. Therefore, the present study sought to identify the factors that contribute to the oxidative cleavage of the polysorbate (PS) molecule and to ascertain the preferred sites of degradation. In order to gain insight into the radical susceptibility of the individual polysorbate segments and their accessibility to water, conceptual density functional theory calculations and molecular dynamics simulations were performed. The behavior of monoesters and diesters was examined in both monomer form and within the context of micelles. The theoretical results were corroborated by experimental findings, wherein polysorbate 20 was subjected to 50 ppb Fe^2+^ and 100,000 lx·h of visible light, and subsequently stored at 25 °C/60% r.h. or 40 °C/75% r.h. for a period of 3 months. Molecular dynamics simulations demonstrated that unesterified polyoxyethylene(POE) chains within a polysorbate 20 molecule exhibited the greatest water accessibility, indicating their heightened susceptibility to oxidation. Nevertheless, the oxidative cleavage of esterified polyoxyethylene chains of a polysorbate 20 molecule is highly detrimental to the protective effect on protein particle formation. This occurs presumably at the oxyethylene (OE) units in the vicinity of the sorbitan ring, leaving a nonamphiphilic molecule in the worst case. Consequently, the critical degradation sites were identified, resulting in the formation of degradation products that indicate a loss of amphiphilicity in PS.

## Introduction

### General Relevance of Polysorbate and Its Degradation

In recent years, biopharmaceuticals have gained considerable importance as they represent many of the best-selling drugs. In 2023, for instance, eight out of the top ten drugs were biologics.^1^ However, the manufacturing processes, transport and/or storage of these products can lead to the formation or degradation of protein particles, which may result in quality issues.^2−7^ Given that proteins are surface-active compounds, contact with hydrophobic interfaces, such as the air–liquid interface, tubing, filter, and vessels, or contact with the primary packaging may result in partial protein unfolding and particle formation.^8−12^ Consequently, surfactants are employed to stabilize therapeutic proteins in aqueous solutions.^13−15^ Polysorbates (PS) represent the most frequently utilized group of surface-active substances for biologics, with polysorbates 20 (Tween 20) and 80 (Tween 80) being the most prevalent.^16−18^

**Figure 1:**
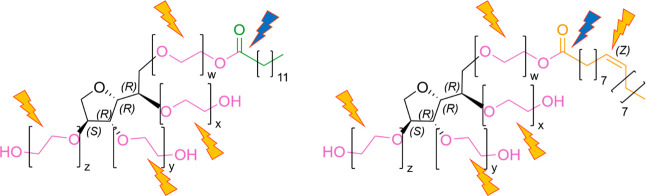
(1) Chemical structure of model polysorbate 20 molecules. The central sorbitan (black) unit is connected to four polyoxyethylene (POE) chains (purple) of variable length with an approximate total chain length of 20 units according to pharmacopoeias.^22,41^*w*, *x*, *y* and *z* were set to 5 OE units within our calculations and simulations. The *w*-chain functions as a connector to a fatty acid (green and orange). Potential radical attacking sites of polysorbate are marked with orange thunderbolts and the hydrolysis of the ester with blue thunderbolts. In the case of polysorbate 20, the most abundant fatty acid is laurate (left), for polysorbate 80 it is oleic acid (right).

Polysorbates are well-tolerated nonionic surfactants with excellent stabilizing properties, a high hydrophilic–lipophilic balance (HLB), and a low critical micelle concentration range (CMR).^2,3,19−21^ Due to their hydrophilic headgroup, comprising a total number of 20 oxyethylene (OE) units (*w* + *x* + *y* + *z*), attached to it and up to four hydrophobic fatty acid tails esterified to the corresponding polyoxyethylene (POE) chains, these substances are highly amphiphilic (Figure 1).^22^ As surface-active substances, they can saturate hydrophobic surfaces in low concentrations and protect proteins from aggregating.^19,23^ Given the significant challenge posed by protein particle formation during production and storage of biopharmaceuticals, it is crucial to implement effective measures to prevent this phenomenon. It should be noted, however, that it is still under debate, how surfactants protect proteins from particle formation. For more details, see Weber et al. (2023).^23−25^

Nevertheless polysorbates exhibit intrinsic stability issues, which manifest at least in part, as particle formation.^26^ The degradation process occurs via two principal pathways, namely hydrolysis and oxidation (Supporting Information Figure 1).^26,27^

**Figure 2:**
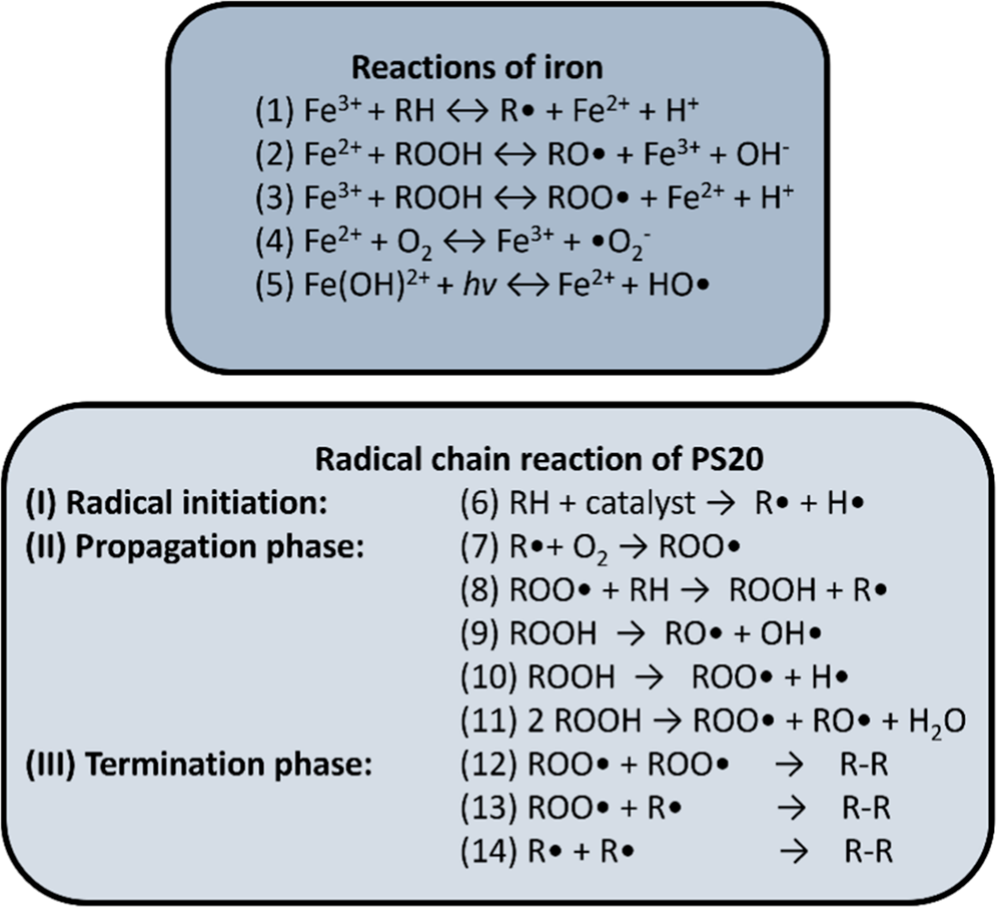
Reactions of iron and radical chain reaction of PS20. Reaction eqs 2 and 3 show the Fenton and Fenton-like reaction, if R corresponds to a hydrogen atom.^53−55^ Reaction eqs 6–14 show the radical chain reaction of PS20 adapted from Donbrow et al. (1978).^27^

Hydrolytic degradation can be subdivided into chemical and enzymatic hydrolysis, which both result in the release of free fatty acids that may form particles.^24,28−32^ Conversely, the sites of degradation during oxidative degradation processes are considerably more diverse due to the presence of multiple potential reaction sites within the PS molecule.^24,33^ Oxidative degradation occurs in three distinct phases, starting with an initiation phase that may be catalyzed by light or iron (reaction eq 6 in Figure 2).^27^ The subsequent radical chain reaction (propagation phase) generates a variety of reactive oxygen species (ROS) (reaction eqs 7–11 in Figure 2).^27^ Iron ions may contribute to this process as they are part of many different reactions generating further ROS (reaction eqs 1–5 in Figure 2). These processes are accelerated through irradiation with light (reaction eq 5 in Figure 2). The formation of reactive oxygen species, including the hydroxyl radical (HO^•–^), superoxide radicals (O_2_^•–^), peroxyl- (ROO^•–^), and hydroperoxyl radicals (HOO^•–^), hydrogen peroxide (H_2_O_2_), hydroperoxide (ROOH) or singlet oxygen (^1^O_2_), has the potential to oxidize the active pharmaceutical ingredient (API) and polysorbates, leading to a loss of function and effectiveness.^34−40^ In order to prevent and implement effective mitigation strategies, it is essential to investigate and gain a comprehensive understanding of the degradation process. Polysorbates are available in a range of esterification grades, including monoesters, di-, tri- or tetra-esters. The corresponding pharmacopoeias specify the different FAs present in these grades.^22,41^ Therefore, this results in a highly heterogeneous mixture, which presents a significant challenge in providing a universally applicable structure.^22^

**Figure 3:**
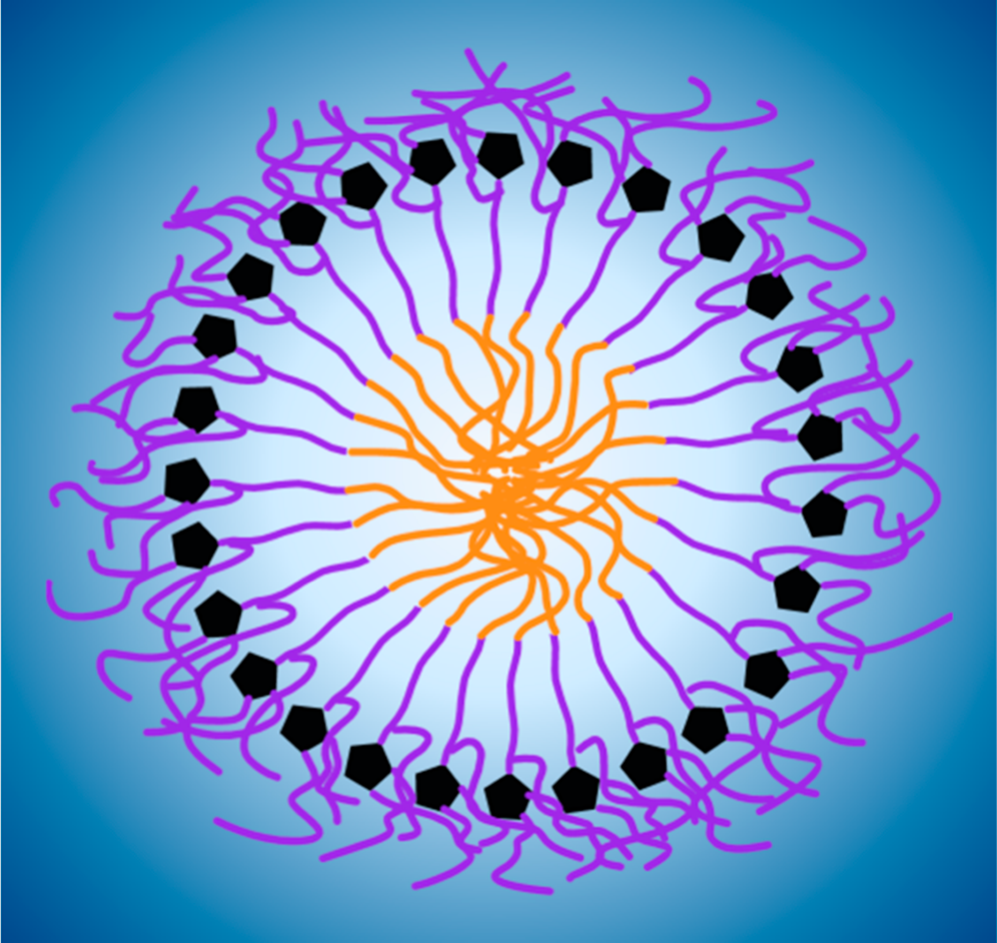
Schematic representation of how the segments constituting a polysorbate micelle in water (blue) are distributed.^51^ The fatty acids (orange) are situated in the center. Moving outward, the “connector” chains (purple) extend to the sorbitan rings (black), linking the two segments together. Lastly, the unesterified POE chains (purple) encapsulate all other segments. The monomers depicted are monoesters, thus featuring just one connector chain. Based approximately on the number of polysorbate molecules comprising a micelle discussed by Ehrit et al. (2023), the micelle consists of 27 monomers, as all micelles do within our simulations.^51^

To understand the oxidative degradation process, knowledge of potential reaction sites is beneficial (Figure 1). Based on a simplified chemical structure of polysorbate 20, in which each POE chain carries the same number of OE units, it is possible to consider different oxidative reaction sites. The prediction of reactivity through the utilization of sophisticated quantum mechanical and semiempirical calculations is on the cusp of becoming routine for larger organic and biological systems.^42−44^ Despite the abundance of research on various polysorbates, there is a dearth of in-depth density functional theory studies and molecular dynamics (MD) simulations examining the stability issues associated with PS20. Consequently, in silico calculations and molecular dynamics simulations were conducted to ascertain the principal sites of oxidative degradation within the polysorbate molecule. Electron structure studies were performed to characterize each atom’s radical susceptibility within a PS molecule. The objective of the MD simulations was to ascertain the accessibility of each polysorbate segment to water and, consequently, to ROS within a micellar environment. The insights from electronic structure calculations can be formulated into chemical language with conceptual DFT (cDFT).^45−50^ A model of a PS molecule was constructed, comprising both mono- and diesters. The behavior of these substances was examined in both monomer and a micellar structure. For the sake of simplicity, both types of polysorbates were esterified with their most prevalent fatty acid, namely lauric acid (C12) for PS20 and oleic acid (C18:1) for PS80. The simulated molecules were composed of 20 OE units, distributed equally across all four POE chains. The micelles simulated in this study were composed of 27 molecules, as experimental studies of polysorbate micelles show that they typically comprise 20 to 50 monomers.^15,51^ A recent computational study by Lou et al. on polysorbate 80 micelles demonstrated that they could be formed by 13 to 61 PS molecules through self-assembly, further supporting our choice.^52^Figure 3 depicts a micellar structure constructed from monomers of a representative PS20 molecule, as PS20 was the focus of the study. It should be noted that simulations utilizing solely monoesters or diesters (as observed in our studies) do not necessarily reflect the actual polysorbate molecules present in biopharmaceutical solutions. Nevertheless, they can offer insights into the potential sites of oxidative degradation.

Subsequently, a comparison was conducted between the aforementioned in silico simulations and the experimental behavior of PS20 following an increase in oxidative stress induced by catalytic Fe^2+^ and light exposure, with subsequent storage at 25 °C/60% r.h. as well as 40 °C/75% r.h. for 3 months. This involved investigating the individual PS segments regarding their oxidative susceptibility using reversed-phase ultra high-performance liquid chromatography system coupled with a quadrupole Dalton based mass spectrometer detector (RP-UHPLC-MS(QDa)).

Ultimately, this will contribute to the comprehension of the preferred locations for solvent and ROS accessibility within the PS molecule, thereby providing insight into the oxidative degradation process within the PS molecule.

## Materials and Methods

### Materials

The solvents employed for UHPLC-MS analyses were acetonitrile (ROTISOLV ≥99.8%, LC–MS grade), which was procured from Carl Roth GmbH (Karlsruhe, Germany). Ammonium formate (≥99.9% LC–MS grade) was purchased from Sigma-Aldrich (St.Louis, MO, USA), while the formic acid (OPTIMA ≥99.0% LC–MS grade) and methanol (OPTIMA ≥99.9% LC–MS grade) were provided from Thermo Fisher Scientific Inc. (Waltham, MA, USA). Milli-Q was obtained from an IQ 7000 Ultrapure Lab Water System from Merck KGaA (Darmstadt, Germany). Sodium chloride was also purchased from Merck KGaA (Darmstadt, Germany). The polysorbate 20 used was of high purity quality (HP) and obtained from Croda (Snaith, United Kingdom). The batch quality and purity profile are within the expected specifications of the European Pharmacopoeia (Ph.Eu., 11th edition) (see Supporting Information Table 1). Iron(II) chloride was procured from Sigma-Aldrich (St. Louis, MO, USA).

### Experimental Methods

#### Reversed-Phase Ultra High-Performance Liquid Chromatography System Coupled with a Quadrupole Dalton Based Mass Detector (RP-UHPLC-MS(QDa))

A UHPLC method was developed based on the approaches described by Lippold et al. (2017)^200^ and Evers et al. (2020).^56^ It employs an Ultimate 3000 UHPLC system, comprising a RS dual gradient pump, RS column compartment and RS autosampler (Thermo Fisher Scientific, Waltham, MA, USA), for all measurements.^56^ The system was coupled to an ACQUITY QDa mass detector (Waters Corporation, Milford, MA, USA), which has an electrospray ionization (ESI) source. A mixed-mode column (Water Oasis Max; 30 μm, 2.1 mm × 20 mm, 80 Å) was used for solid-phase extraction. For separating all PS subspecies, a reversed-phase column, a Poroshell 120 SB-C8 4.6 × 100 mm, 2.7 μm (Agilent Technologies, Inc., Santa Clara, CA, USA) was employed as the stationary phase. The analytical gradient consisted of three mobile phases (A: 100% acetonitrile, B: 100% ultrapure water, C: methanol). A T-piece mixed a 10 mM ammonium formate buffer, delivered by the left pump at a rate of 0.2 mL·min^–1^, with the analytical gradient, delivered by the right pump at a rate of 0.7 mL·min^–1^, after the PS subspecies had been separated by the stationary phase. The column oven was set to 50 °C for the analysis of PS20, and an injection volume of 2 μL was employed. The run time was 45 min with the QDa detector operating in positive mode to analyze masses between 250 and 1250 *m*/*z*. The sampling rate was set to 2 Hz. Standards of PS20 were prepared and measured, with a range of 0.05 to 0.6 mg·mL^–1^ and a limit of quantification of (LOQ) of 0.05 mg·mL^–1^.

A discussion was required regarding the charges to be considered for the respective PS20 species. In general, the method used is capable of detecting single, double and triple charges. The measured *m*/*z* ratios, along with the corresponding charges, including their intensity as well as their retention time were exported from Empower for subsequent analysis with the support of an algorithm. The measured *m*/*z* ratios were then compared with the masses of known degradation products, and the ratios were assigned according to their mass. In doing so, the retention time is also taken into account.

The general nomenclature is described in Table 1. For all POE-FA (and isosorbides), an evaluation was conducted utilizing the single-charged *m*/*z* ratios, as minimal intensity was observed in the double-charged POE-FA. In contrast, the single-charged species were identified as having the highest intensities and were therefore used for subsequent analysis. Conversely, all sorbitans, i.e. all sorbitan rings with corresponding POE units plus esterified fatty acid (with the exception of S-00++, where the sorbitan is provided with POE but without esterified fatty acid), were analyzed using the double-charged species, since these species with single- or triple-charged *m*/*z* ratios fall outside the range of the measurement method. Consequently, they are most frequently detected as double-charged species.

**Table 1:**
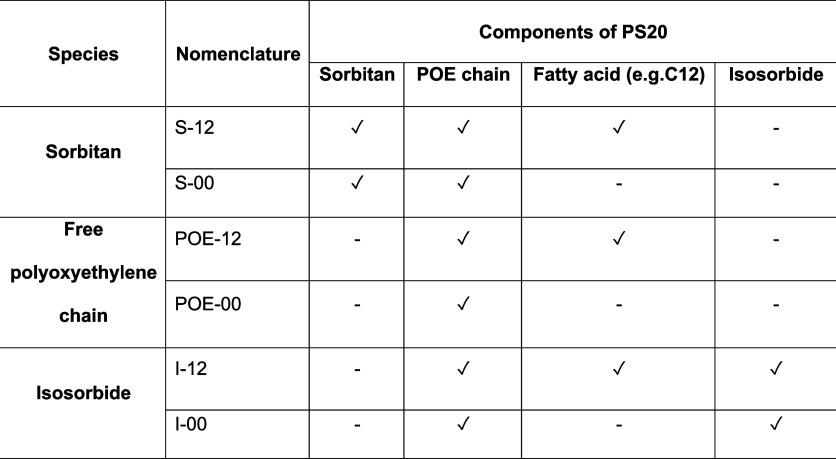
Nomenclature of Polysorbate (PS20) Species Determined via UHPLC-MS Measurements. PS20 Consists of Sorbitans with or without Fatty Acids, Free Polyoxyethylene (POE) Chains with or without Fatty Acids and Isosorbides with or without Fatty Acids^a^

a Free POE chains may be initial impurities or degradation products.

**Table 2:** Model Polysorbate Molecules Used for Molecular Dynamics (MD) Simulations of Monomers and Micelles in Water

fatty acid	esterified POE chain	monomer count	denomination
lauric acid	*w*	27	monolaurate
oleic acid	*w*	27	mono-oleate
lauric acid	*w*, *x*	27	dilaurate

For these studies, including all charges does not appear appropriate, as the most probable (+ or ++) form of charge may be influenced by the other two forms of charge. Accordingly, the decision was taken to evaluate the most frequently occurring charge form.

#### Formulation of Polysorbate Samples

To investigate the oxidative degradation of PS molecules as well as to determine the degradation products, a stress study was conducted using Fe^2+^ and 100,000 lx·h as the initial stress factor. The initial step involved the preparation of a stock solution that was prepared in Milli-Q water to obtain a 50 ppb Fe^2+^ solution. Prior to utilization, the solution was sterile filtered using a 0.22 μm syringe filter, in a manner analogous to that employed for the 100 mg·mL^–1^ PS20 stock solution. The PS20 stock solution and the Fe^2+^ stock solution were spiked together, resulting in a solution with 0.4 mg·mL^–1^ (163 μM) PS20 and 50 ppb Fe^2+^, with a pH of 6.3. Subsequently, the vials were sealed with rubber stoppers and capped with 20 mm aluminum caps. The vials were irradiated with 100,000 lx·h for 17 h, with temperature control maintained at 25 °C and a relative humidity of 60% r.h. using a climate chamber, the PharmaEvent C/500L from Weisstechnik (Balingen-Frommern, Germany).

#### Computational Methods

Besides employing the above-mentioned experimental techniques, we also employed computational methods to investigate polysorbates. These include molecular dynamics (MD) simulations and (conceptual) density functional theory (c)DFT calculations as outlined below. Given the complexity of polysorbate mixtures already mentioned in the introduction, it was necessary to select representative model systems for the computational investigation. The rationale behind this selection is outlined in the following section.

#### Investigated Systems

As PS is a very heterogeneous mixture, simplified PS models were selected for the present analyses. We focused on two key variables that characterize such polysorbate compounds, (i) the type of fatty acid ester and (ii) the degree of esterification (Table 2). In the case of the diester, we also probed the role of the esterification site (Supporting Information Figure 2). To further reduce the complexity, all POE chains were set to be composed of 5 OE units in every system (Figure 1). Moreover, each simulation with more than one molecule comprised only one type of model polysorbate system, avoiding the increased complexity of mixed systems.

#### Molecular Dynamics Simulations

For each system outlined in Table 2, the following system preparation was employed. First, the structure of the respective polysorbate monomer was constructed in Maestro (13.9.135) based on the 1,4-sorbitan structure provided by the Cambridge Structural Database,^57^ ensuring that it possessed the most prevalent stereochemistry of the sorbitan ring according to the polysorbate synthesis described by the Ph.Eur., i.e., the 1,4 stereoisomer.^22^ The structure was then parametrized in Maestro with the OPLS_2005 (Optimised Parameters for Liquid Simulations) force field. The resulting Desmond parameter file was converted to a GROMACS topology and structure file to be compatible with the chosen simulation engine using the “intermol convert” program and some manual intervention (details to the adaptation of the.cms file to.top &.gro in Supporting Information).^58^

For every simulation conducted using GROMACS (2020.2) with the OPLS-AA force field,^59−61^ the initial structures (as prepared with Maestro, see above) were solvated in a cubic box of approximately 36,000 TIP4P-EW water molecules and approximately 105 Å in side-length. Periodic boundary conditions were utilized in all directions. Electrostatic interactions were treated with fast smooth particle-mesh Ewald (SPME). The simulation protocol was composed of an initial energy minimization, a 500 ps temperature equilibration at 300 K in the canonical ensemble (*NVT*), a 500 ps pressure equilibration at 1 bar in the isothermal–isobaric ensemble (*NpT*) and finally a 1 μs production run also in the *NpT* ensemble. For further details please refer to the Supporting Information.^62^

The described simulation protocol was used to simulate the individual monomer molecule of the respective system in water. Since polysorbate molecules self-assemble to micelles at concentrations typically found in pharmaceutical formulations, our objective was also to simulate the interactions of multiple PS molecules and micelle formation. Consequently, 27 random conformations of PS20 molecules in water boxes were extracted from the respective individual monomer simulation. These were then used to construct a 3 × 3 × 3 cubic arrangement comprising 27 solvated polysorbate molecules (Table 2). To ensure that there were no steric clashes, each molecule was separated according to the most stretched-out conformation (details in Supporting Information). The cube of monomers was then simulated to observe micelle self-assembly. In all micelle simulations presented here, the initial 200 ns of the production run were excluded from the data that was used for the analyses below as micelle self-assembly took place in the initial part of the simulation. Following the formation of the micelle, in each simulation, the morphology of the micelle remained very stable with no large-scale rearrangements. This can be observed from the radius of gyration of the simulated micelles, which demonstrate minor fluctuations around a steady value (Supporting Information Figure 3), thus characterizing the sampling portion of the simulation.

#### Quantum Chemical and Electronic Structure Calculations

Tight-binding theory was applied to determine the radical susceptibility in terms of the Fukui index. The Fukui index is closely related to the frontier molecular orbital (FMO) theory, which is used to identify locations in a molecule that are susceptible to electrophile, nucleophile and radical attacks.^45−50^ The Fukui index represents a refinement of the single reference used in FMO, accounting for the change in electronic density with a surplus and a deficit of an electron. The tight-binding calculations were employed in xTB; the atom-wise partial charges were evaluated with the Hirshfeld partitioning method in MultiWFN.^63−67^

#### Water Contacts Analysis

The molecular dynamics simulations were analyzed using Python programs based on the MDAnalysis and Numpy libraries, with the simulations themselves being performed and processed with GROMACS.^68−70^ During a simulation, the fully assembled micelle may travel through the periodic box. To ensure consistency in the subsequent analysis, the micellar structures were unwrapped in accordance with the minimal image convention as part of the processing. Thus, the entire micelle could be analyzed.

At relevant concentrations within biologics, polysorbates predominantly form micelles. To identify potential sites of oxidative degradation, we analyzed the solvent accessibility of various PS micelles in water. Polysorbate molecules were divided into components (e.g., fatty acid, POE chains, sorbitan), and the number of water molecules close to each segment was counted during the simulation and divided by the frame count. These analyses suggested that connector POE chains were crucial for polysorbate oxidation. We therefore also analyzed the water accessibility of each atom within the connector chains, in a manner analogous to that employed for the segments. A more detailed explanation of this calculation is given in the Supporting Information.

## Results

First, computational analyses on simulations with PS20 and PS80 are shown. Subsequently, the results of the practical laboratory experiments with PS20 in the presence of Fe^2+^ and 100,000 lx·h light, which act as oxidative stressors, are presented. To facilitate the presentation of results, mono- and diesters were simulated with the most abundant fatty acid (FA) present in each case: lauric acid (C12) for PS20 and oleic acid (C18:1) for PS80. All fatty acids were esterified to the *w*-POE chain, or, in the case of diesters, additionally to an additional POE chain (*x*, *y* or *z*) (Table 2). All POE chains of the model systems were composed of 5 OE units, with the 20 OE units specified by the Ph.Eur. distributed equally among all four POE chains. The number of molecules in the polysorbate micelles was set to 27 in order to facilitate the computational setup and reduce the simulatalion time, while maintaining a plausible range, given that PS micelles have typically been observed to contain between 20 and 50 monomers.^15,51^

### Density Functional Theory Calculations—Radical Susceptibility of Polysorbates

The reactivity of a molecule can be described by conceptual DFT (cDFT). In order to achieve our objective of elucidating the reactivity of each atom within a PS molecule based on the experimental degradation pattern, we have elected to focus on the local descriptor for radical susceptibility. The following questions were addressed: (i) whether there is a difference in local reactivity, (ii) how it changes upon conformational changes and (iii) whether there are differences between polysorbate 20 (Tween 20) and polysorbate 80 (Tween 80). The results of our calculations indicate that the conformational space is not a determining factor in the radical susceptibility of the POE chains.

**Figure 4:**
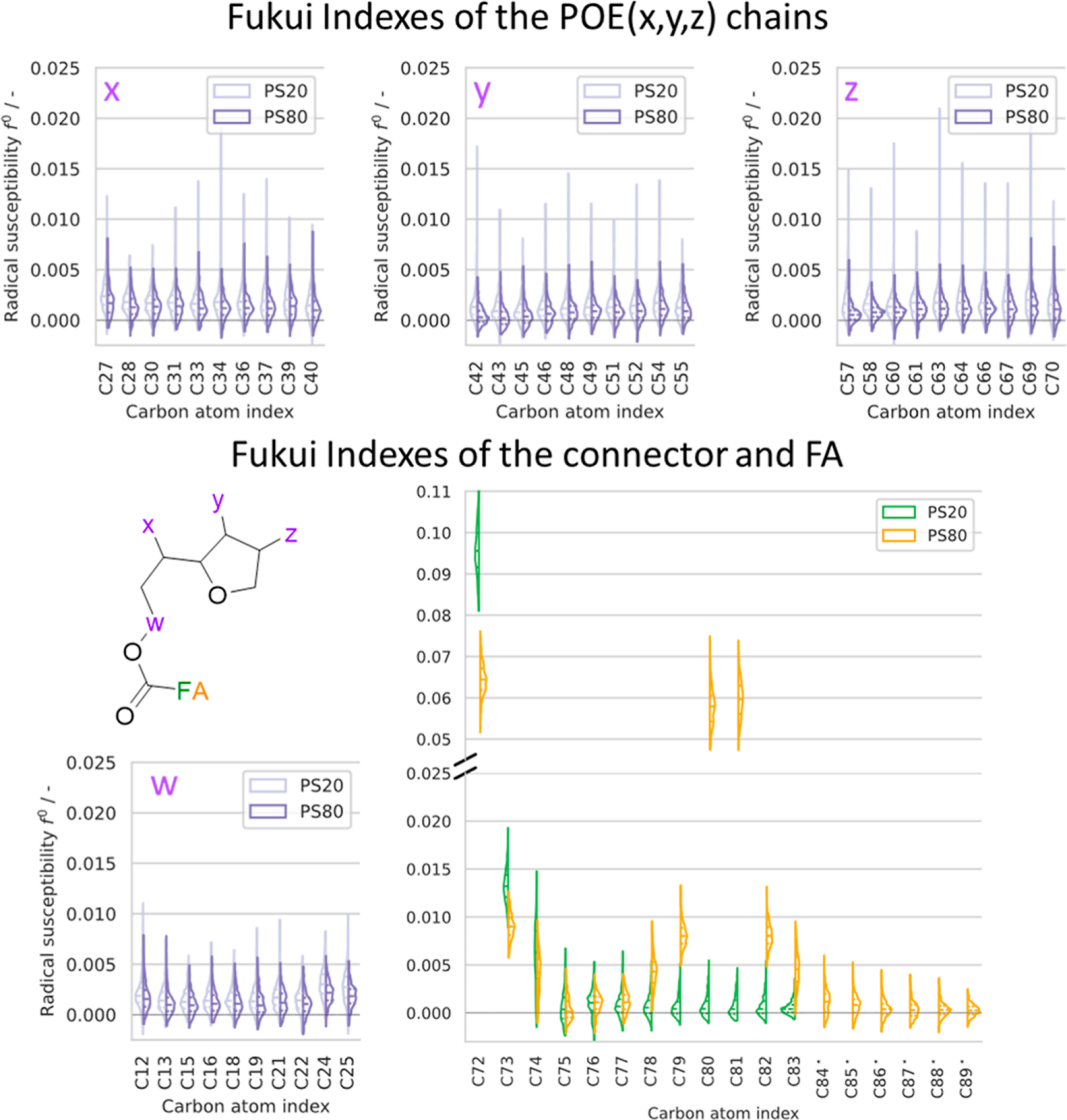
Fukui index *f*^0^ distributions (xTB and Hirshfeld partitioning method) based on 500 conformations from the MD simulations of monomers of polysorbate 20 (with lauric acid esterified) on the left-hand (light violet and green) and monomers of polysorbate 80 (with oleic acid esterified) on the right-hand (dark violet and yellow) violin-halves. The distribution of C72 (carbonyl carbon atom) of both polysorbates and the C80 and C81 of PS80 (carbon atoms of the double bond of the oleic acid) are a magnitude bigger than the presented *f*^0^ of sorbitan and the polyoxyethylene units. C84*-C89* represent carbon atoms within oleic acid (PS80) that are not part of lauric acid (PS20).

The Fukui indexes (FI) of polysorbate 20 and 80 monomers were derived from 500 frames of the classical molecular dynamics (MD) simulations and are presented in violin plots in Figure 4. Given the absence of notable discrepancies, the outcomes pertaining to free PS monomers are presented in the subsequent figure (Figure 4). The *x*-axis shows the carbon atom identifier within the polysorbate molecule, while the *y*-axis displays the radical susceptibility *f*^0^. Figure 4 is divided into sections representing the different segments of PS (*w*, *x*, *y*, *z* POE chains and the fatty acid). All POE chains are depicted in light violet for PS20 and dark violet for PS80. The sum of all calculated Fukui indexes is equal to 1.0.

The most noticeable atoms include C72 (carbonyl carbon) as well as C80 and C81 of the oleic acid. The carbonyl carbons (C72) of lauric and oleic acid exhibit mean values of 0.096 and 0.065 respectively. Additionally, the *f*^0^ of the sp^2^-hybridized carbons (C80 and C81) in the oleic acid distribute around 0.058 and 0.060 (Figure 4). As these have shown a much broader range of distribution, they are displayed with an increased width to improve visual clarity (factor 3).

The Fukui indexes of the esterified polyoxyethylene units (POE) are found to be uniform with values slightly above 0 (*w* in Figure 4). A comparable outcome was observed for the Fukui indexes, pertaining to the unesterified POE arms (*x*, *y*, *z*), which also showed a homogeneous pattern for both PS20 and PS80, with values slightly above 0 (Figure 4). Detected deviations in the most abundant Fukui index lay inside the wide distribution of all carbons of each branch. Therefore, no significant distinction was observed between the individual POE chains. Similarly, minimal variation was discernible within a POE chain.

Additionally, FI calculations were employed for a reduced PS model (Supporting Information Figure 7) to investigate the impact of implicit solvent models (water and ether) on a conformational space search (Supporting Information Figure 8).

In general, only minor differences were noted with regard to the local reactivity (C72, carbonyl carbon). Comparing PS20 and PS80 revealed an increased radical susceptibility within the unsaturated fatty acid of PS80 (C80/81). Nevertheless, based on FI calculations no differences regarding the radical susceptibility of the POE chains were observed.

### Molecular Dynamics (MD) Simulations

#### Solvent Accessibility

To identify potentially favored oxidation sites, we performed a series of simulations of micelles in which we investigated polysorbate segments for their solvent accessibility. At concentrations commonly used in biopharmaceuticals (0.01–1 mg·mL^–1^), PS is often used above its critical micelle concentration range (CMR) (0.018–0.09 mg·mL^–1^ for PS20, 0.009–0.021 mg·mL^–1^ for PS80), so micelles are present.^15,24^ Yet, PS is of course not only present as micelles even above the CMR, in fact, monomers and micelles are in equilibrium.^71−74^ As mentioned, all micelle simulations were set up with a cubic arrangement of 27 evenly spaced polysorbate molecules. The observed micelle self-assembly is essentially the same for each simulated system, except for the exact time it takes. Self-assembly occurred in every simulation performed without exception within at most 250 ns or less of the simulation time, but usually before reaching 200 ns. The assembly times can be assessed in Supporting Information Figure 6. During the assembly process, the polysorbate molecules typically move closer together until they form a cluster. Many such clusters can form simultaneously and then merge into larger ones, which also requires a rearrangement of the polysorbate molecules, since the fatty acids have a very strong tendency to be protected from the water in the core of the micelle (Supporting Information Figure 3). Such a process is entropy driven due to the hydrophobic effect.^75,76^ Alternatively, single polysorbate molecules can also be incorporated into existing clusters. When these two processes are combined, after a short time (250 ns at most) a micelle comprising every polysorbate molecule in the simulation is observed. The remaining simulation after micelle self-assembly was used for all further analyses. The average radius of gyration over the 800 ns of sampling period for the assembled micelle is 19.3 Å for the *w*-lau micelle, 19.7 Å for the *w*-ole micelle and 20.1 Å for the *wx*-lau micelle.

In order to better understand the structure of polysorbate micelles, the polysorbate molecule has been divided into segments according to their chemical properties, resulting in a separation into the following groups: the fatty acid, the sorbitan ring (SOR) and the POE chains (*w*, *x*, *y*, *z*-POE) (Figure 1). The latter can be further subdivided into POE chains that connect the sorbitan to a fatty acid (hereafter referred to as “connector” chains) and the unesterified POE chains that are only attached to the sorbitan. For each system investigated (*w-lau*, *w-ole*, *wx-lau*), the radial distribution function of the atoms in each segment with respect to the center of mass (COM) of the micelle was calculated with a Python program based on the MDAnalysis library, showing how far each segment is distributed from the core of the micelle. A schematic representation of a polysorbate micelle according to the results can be seen in Figure 6 (detailed comparison in Supporting Information Figure 3). We found that the fatty acids form the core of the micelle, followed by the connecting POE chains, which are the next-closest segments to the COM. The sorbitan ring follows and finally the unesterified POE chains are found furthest from the center of mass encapsulating all other segments and interfacing with the water.

We hypothesized that reactive oxygen species found in water would be the main culprit of degradation.^77,78^ Since the rate of oxidation by ROS is directly influenced by the solvent accessibility of specific segments, the latter serves as a practical approximation for the radical accessibility on a PS20 molecule.^79^ Thus, for each system investigated, the water contacts were computed (calculation details available in the Supporting Information) to determine the most likely oxidation sites of the polysorbate molecule.

**Figure 5:**
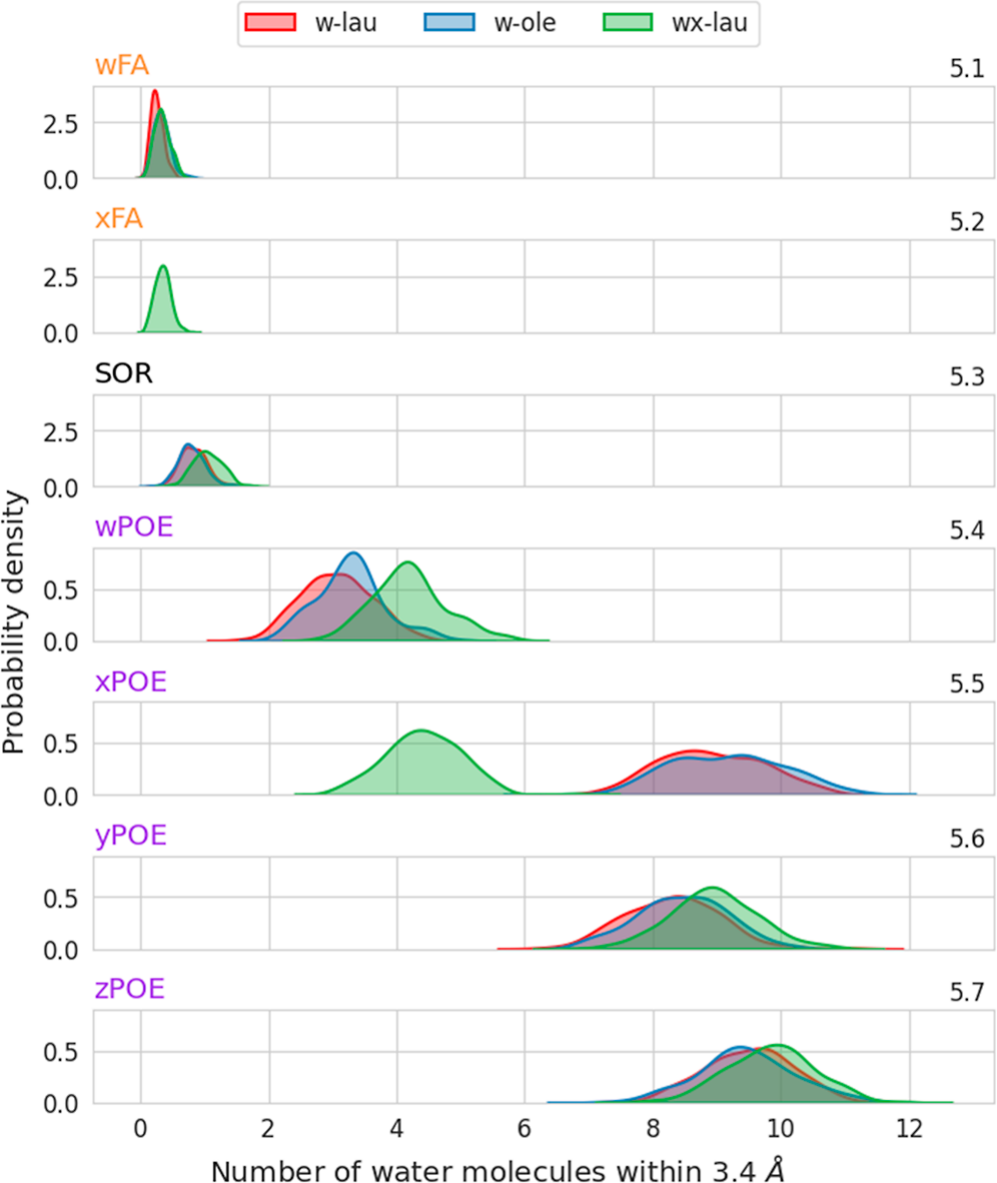
Probability of contact with water for each segment of a polysorbate molecule in a micelle. Each subplot is titled according to the respective polysorbate segment displayed and color-coded as depicted in Figure 1. The distributions were constructed out of the respective contacts time series data, making differences in probabilities easier to identify. The systems portrayed include the all-laurate monoester shown in red, the all-laurate diester in green, and the all-oleate monoester in blue. 3.4 Å was used as contact cutoff, as this encloses the distance between the heavy atoms in a hydrogen bond with an additional small margin.

**Figure 6:**
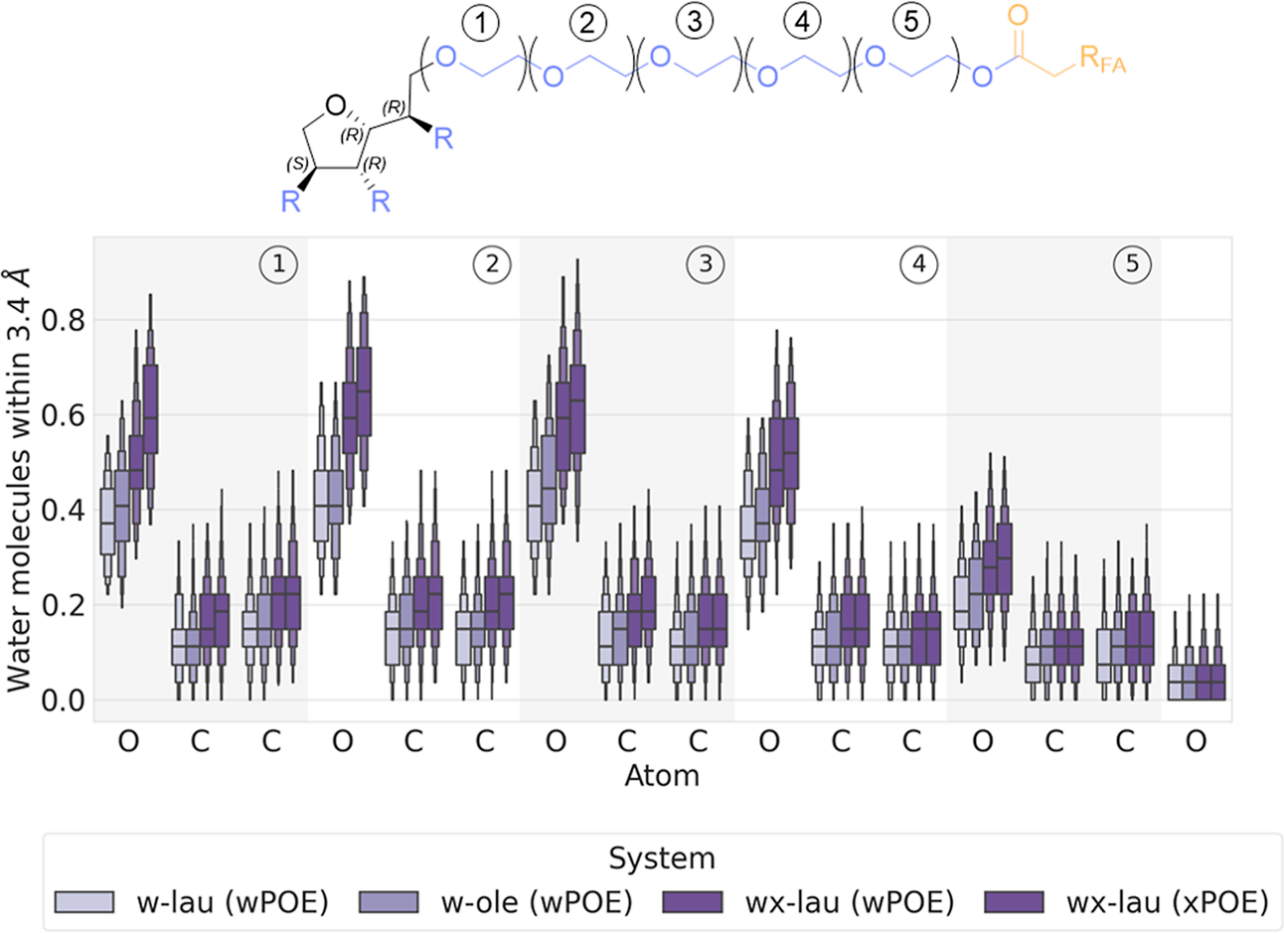
Number of contacts with water for each atom of the connector segment (esterified POE chain (at *w*- or *x*-POE)), arranged from left to right, starting from the sorbitan ring and progressing toward the fatty acid (see numbers of the structure above the plot). Contact distributions for the connector chains of three systems are displayed for each atom of the segment. Color coded with progressively darker shades of purple, from left to right for each atom, the laurate monoester (*w*-lau (*w*POE)), oleate monoester (*w*-ole (*w*POE)), and laurate diester (*wx*-lau (*w*/*x*POE)) chains are displayed. The laurate diester, with two connector chains, exhibits two distributions for each atom (*wx*-lau (*w*POE) and *wx*-lau (*x*POE)). Alternating shaded blocks highlight individual OE units of the POE chain, each labeled with a number from 1 to 5. The most right oxygen atom (O) refers to the ester group oxygen.

Whether the micelle consists of all-laurate monoesters (*w*-lau), all-oleate monoesters (*w*-ole) or all-laurate diesters (*wx*-lau) has no statistically significant effect on the contacts of the fatty acids with water (Figure 5, especially 5.1 and 5.2, *w*-FA and *x*-FA). As can be gathered from Supporting Information Figure 6, no water is present in the hydrophobic core of the micelles at any time after they have assembled. The nonzero amount of water contacts in Figure 5 is exclusively due to contacts of water with the fatty acid that arise when water reaches the layer of the micelle surrounding the fatty acid core. Inspecting the solvation of the sorbitan segment (SOR) no difference is found between monoesters of different fatty acids (*w*-lau and *w*-ole) (Figure 5.3). In contrast, the introduction of a second ester (*wx*-lau) significantly increases the contacts with water for the sorbitan ring (from about 0.8 to 1.1) compared to the monoesters (*w*-lau and *w*-ole) (Figure 5.3). All systems (*w*-lau, *w*-ole, *wx*-lau) shown in Figure 5 have a fatty acid attached to the *w*-POE chain, making it a connector. The latter shows a count of contacts with water that is lowest for the monoester with the shortest fatty acid (*w*-lau), increases significantly when the fatty acid is more sterically demanding (*w*-ole) and is highest for the diester (*wx*-lau) (3.1, 3.3, 4.2 contacts on average respectively) (Figure 5.4, *w*-POE). Only for the all-laurate diester system (*wx*-lau), with two fatty acids on each molecule, the *x*-POE chain is also a connector, as is the *w*-POE chain in all shown systems. The *wx*-lau *x*-POE chain is more shielded from the solvent than the unesterified *x*-POE chains of the *w*-lau and *w*-ole monoesters (Figure 5.5). This shielding results in a lower solvation of 4.4 contacts for the diester (*wx*-lau) compared to a much higher number of contacts for both monoesters (8.9 for *w-lau* and 9.2 for *w-ole*), which correspond to a free *x*-POE chain and exhibit equal solvation (Figure 5.5, *x*-POE). The *y*- and *z*-POE chains are not esterified in any of the systems shown. As such, their number of water contacts is equal between the *wx*-lau and *wx*-ole monoesters (8.3 *w-lau y*-POE, 8.5 *w-ole y*-POE (Figure 5.6); 9.5 *w-lau z*-POE, 9.5 *w-ole z*-POE (Figure 5.7)). In case of the diester, an equal distribution to the monoesters is observed for the *z*-POE chain (9.8 *wx-lau z*-POE vs. 9.5 *w-lau z*-POE (Figure 5.7)), while the *y*-POE chain differs with 9 *wx-lau y*-POE vs. 8.3 *w-lau y*-POE contacts (Figure 5.6).

In general, for the same connector POE chain (*w*-POE), the monoester with the shortest fatty acid (*w*-lau) has the lowest number of water contacts. This number increases for the monoester with a more sterically demanding fatty acid (*w*-ole) and is highest for the diester (*wx*-lau) (Figure 5.4). A connector, i.e., an esterified POE chain such as the *x*-POE of the *wx*-lau, shows fewer contacts with the solvent than the corresponding unesterified POE chains such as the *x*-lau or *x*-ole. In conclusion, the connector chains seem to show the highest system-dependent variability in our simulations (Figure 5, especially 5.4 and 5.5, *w*-POE, *x*-POE).

#### Solvent Access to the Connector Chain Atoms

As the major discrepancy in water exposure between the same segment of the three analyzed systems (the all-laurate mono- and diester as well as the all-oleate monoester) was observed at the connector chains (Figure 5, especially 5.4 and 5.5, *w-POE*, *x-POE*) the heavy atom water contacts were calculated for each atom of the connector chains.

The first OE unit of the *w*-connector chain shows a substantial difference in the contact probability between the diester POE chains (dark violet) and the monoester POE chains (lighter violet) of each atom within the OE unit (Figure 6.1). The oxygen atom, which is the most likely to be involved in hydrogen bonding, has a higher probability of being close to water than the adjacent carbon atoms. For all three atoms of the first OE unit, the diester distributions (*wx*-lau (*w*/*x*POE)) show a higher probability of water contact than the monoesters (*w*-lau and *w*-ole) (Figure 6.1). The difference between the latter is smaller. The carbon atoms show very similar distributions, but the oxygen atom of the *w*-ole monoester is noticeably more in contact with water (0.41) than the same atom of the *w*-lau monoester (0.38). The diester distributions are much further apart from the monoester distributions, with 0.50 for the *wx*-lau (wPOE) chain oxygen and 0.59 for the *wx*-lau (xPOE) chain (Figure 6.1).

The second, third and fourth OE units display very similar water contact probabilities of the carbon atoms as those in the first OE unit (Figure 6.1–6.4). For the oxygen atoms, the same trend of the first OE unit is reflected in the other OE units, namely that the diester atoms have a consistently higher propensity to solvate than the monoester atoms. Among the monoesters, the difference is again smaller than between a monoester and the diester. Except for the second OE unit, where the distribution of the *w*-lau and *w*-ole oxygens is very similar, the monoester with the larger fatty acid (*w*-ole) also has a consistently slightly higher water contact probability at each OE oxygen atom than its equivalent with the smaller fatty acid polysorbate (*w*-lau).

The highest water contact probabilities of the whole connector chain are found at the first, second and third OE units within the oxygen atoms for all systems analyzed (*w*-lau −0.41 (Figure 6), *w*-ole −0.46 (Figure 6.3), *wx*-lau (wPOE) −0.59 (Figure 6.3) & (xPOE) −0.64 (Figure 6.2)). The fourth OE unit shows decreasing contact probabilities compared to the third OE unit (Figure 6.2–6.4).

The fifth and final OE unit has the lowest water contact probability of all the connector oxygens for each system. The difference between the oxygen probabilities of the diesters (*wx*-lau) and monoesters (*w*-lau, *w*-ole) is also less pronounced compared to the other OE units. The carbon–water contact probabilities of all systems in the fifth OE unit are also very comparable, with the exception of *w*-lau (*w*POE), which shows significantly lower values (Figure 6.5). The ester alkyl oxygen (O6) shows very similar distributions for all systems which are the lowest contact probabilities of all connector chains.

In summary, Figure 6 shows a general trend for the oxygen atoms in the connector POE chains with an average water contact probability about three times higher than that of the carbon atoms of the same chain, as expected given the hydrogen bonding capabilities of the former. The oxygen atoms of the diester (*wx*-lau) connectors are consistently more likely to be in contact with water than those of the monoesters (*w*-lau, *w*-ole). The monoester oxygen water contact probabilities of the system with the larger fatty acid (*w*-ole) are always lower than those of the diester, but always higher than those of the monoester with the smallest fatty acid (*w*-lau), following the trend already seen in the segment-wise water contacts (Figure 5). Finally, the ester alkyl oxygen is very unlikely to be exposed to water compared to any other oxygen in the connector chain in all systems.

### Laboratory Experiments

#### Iron Stress Study

The study carried out examines the influence on the oxidative degradation of PS20 after exposure to 50 ppb Fe^2+^ and 100,000 lx·h in a practical laboratory experiment to investigate the practical behavior in comparison with the simulations performed with PS20.

In general, polysorbates consist mainly of sorbitans (about 72%), which may be esterified either once or up to four times (Figure 1). Due to the manufacturing process, impurities in form of free POE chains (esterified or nonesterified) (approximately 8%) as well as isosorbides (approximately 20%) are present from the beginning. However, since polysorbates are defined within the pharmacopoeias as “a mixture of partial esters of fatty acids, [···], with sorbitol and its anhydrides [···]”, mainly the sorbitans and their degradation products, the free POE chains, will be discussed in this paper.^22,41^ Nevertheless, it should be noted that the isosorbides are also degraded at their esterified fatty acids and thus ultimately contribute to the increase of free POE chains (see Supporting Information Table 2).

To evaluate the mass spectrometry (MS) results, the total intensity for all identified species was summed up. For each individual species, the percentage of the total intensity was then determined in order to observe the proportional shift of the total intensity under different storage conditions over time.

The distribution of the OE chain length within the sorbitan species is Gaussian normal and ranges from 0 to approximately 50 OE units distributed over the entire PS20 molecule (depending on the fatty acid chain length and degree of esterification as *m*/*z* ratios from 250 *m*/*z* on are measured). Another interesting aspect is that the sorbitans connected to an esterified POE chain initially carry mostly approximately 24 OE units over the entire molecule, i.e., 4 OE units more than required by the pharmacopoeias.^22,41^ This is irrespective of whether they are mono-, di- or triesters. It should be noted that as the fatty acid chain length increases, shorter OE chains ethoxylated to the molecule are also detected, as for example, from a fatty acid chain length of C12 (S-12++) the *m*/*z* of 250 *m*/*z* required for detection is already achieved with 3 OE units being present (257 *m*/*z*). In contrast, if stearic or oleic acid is esterified (C18 or C18:1), even a single OE unit is detected due to the mass difference between C12 and C18 (257 *m*/*z* for C12 with 3 OE units vs. 255 *m*/*z* for C18 with 1 OE unit). However, as the *m*/*z* is then also limited upward using our method, a triester such as S-12/12/16++ will only be able to detect 38 OE units. Nevertheless, the majority of the species fluctuates in a range of approximately 22–26 OE units (normally distributed). These results show the heterogeneity of the PS raw material. When stored at 40 °C for 3 months, the number of OE units is reduced from 24 to 20 for all di- and triesters as well as for monoesters from C14 up to C18:1.

**Figure 7:**
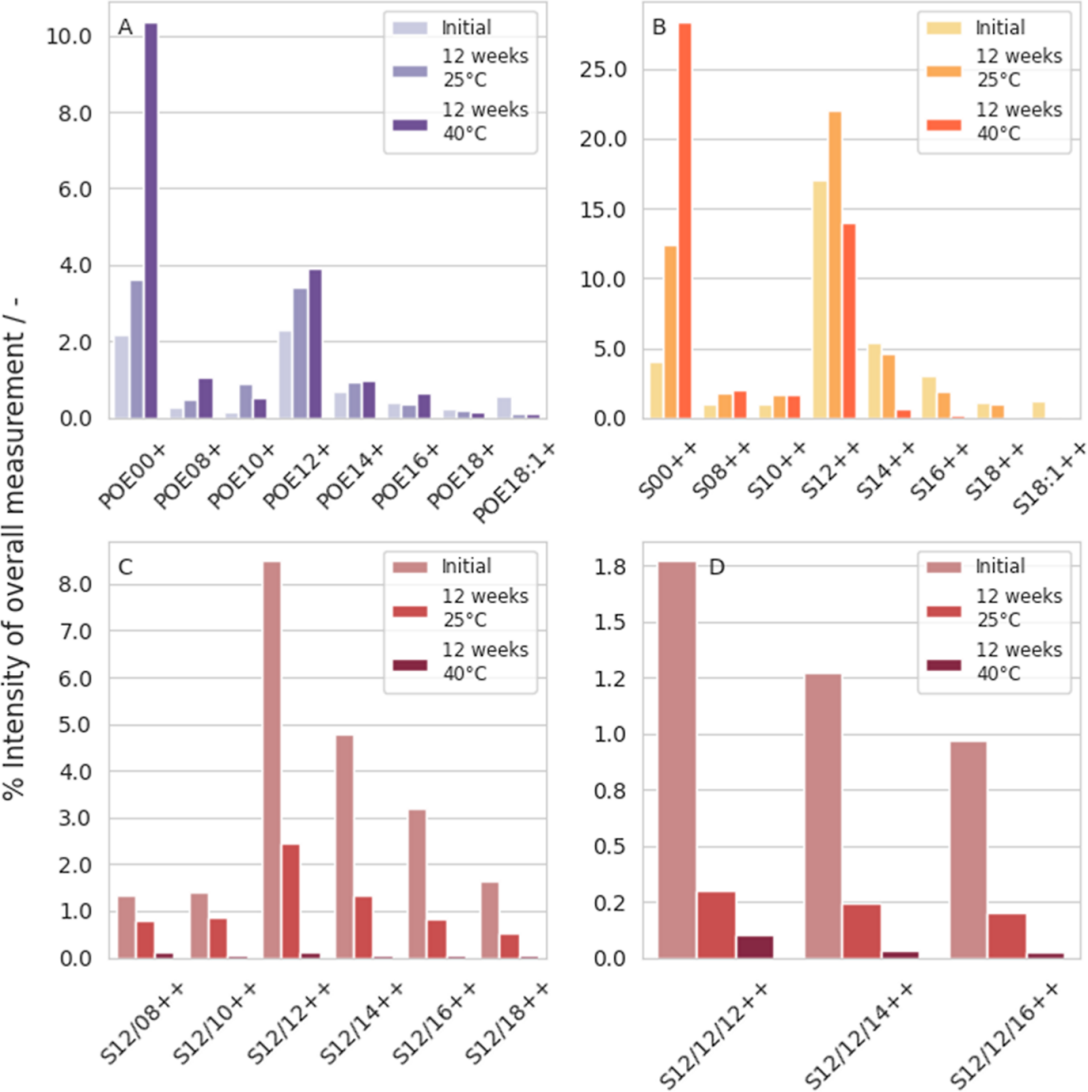
Results of UHPLC-MS analysis of PS20 initially (left column) and after storing at 25 °C (middle column) and 40 °C (right column) for 3 months with 50 ppb Fe^2+^ and 100,000 lx·h light irradiation. After UHPLC-MS analysis different segments of the PS20 molecule are depicted. POE units with esterified fatty acids (POE-fatty acid+ (POE-FA+)) are shown in A. Monoester are shown in B with (sorbitan-fatty acid++ (S-FA++)). Diester are shown in C (sorbitan-fatty acid++ (S-FA/FA++)), triester are shown in D (sorbitan-fatty acid++ (S-FA/FA/FA++)).

POE-*XX*+ indicates that all POE chains with different amounts of OE units and the corresponding fatty acid chain length (*XX*) are summed up together. This distribution is also Gaussian normal. The MS measurements showed that most of the POE units with an attached fatty acid chain (POE-FA-*XX* (chain length)) consist mainly of POE-12+ (Figure 7A). Since lauric acid (C12) represents most of the fatty acids within PS20 (according to the pharmacopoeias), this was not unexpected. POE-12+, for instance, means that POE chains esterified to a C12 chain are a sum of POE chains with 1 OE unit, 2 OE units, 3 OE units and so on, up to 22 OE units in this case.^22,41^ The most common OE chain length is initially observed to be 12 OE units. Therefore, the OE chain length of the free POE-*XX*+ species is on average much larger than in the sorbitan bound forms. The most common number of OE units for all other free POE chains is similarly with 11–13 OE units. Our experiments have also demonstrated a dependence on the fatty acid chain length for the free POE chains, with higher degradation with increasing fatty acid chain length. The 12 OE units of POE-18:1+ decrease by 96% after 3 months at 25 °C, POE-16+ by 71%, POE-12+ by 69% and POE-08+ by 51%. As a result, POE-12+ shows mostly 5 OE units after 3 months at 40 °C (Supporting Information Figure 4).

#### Stressing Polysorbate 20 (PS20) with Fe^2+^ and 100,000 lx·h at Elevated Temperature

Spiking a sample in the presence of 50 ppb Fe^2+^ and 100,000 lx·h initially shows about 2% POE-12+ (Figure 7A). After storing the sample for 3 months at 25 °C an increase to about 3% is already visible, at 40 °C storage the effect is a little more obvious, the POE-12+ increases to about 4% after 3 months (Figure 7A and Supporting Information Table 2). The POE-00+ also increase from about 2% initially to about 4% after 3 months at 25 °C and finally to even 10% after 3 months at 40 °C (Figure 7A and Supporting Information Table 2). The proportion of monoesters in the total intensity is highest for S-12++ with 17%, followed by S-14++ with about 5% and S-00++ with an initial value of 4% (Figure 7B). After storing for 3 months at 25 °C, the monoesters of S-12++ increase to 22% and S-00++ to just over 12% (Figure 7B and Supporting Information Table 2). The monoesters S-14++/S-16++/S-18++ and S-18:1++ decrease between 15 and 90% with increasing degradation rates with longer fatty acid chains except for S-18++ after storing for 3 months at 25 °C. Thus, the monoesters of the longer fatty acid chains (S-16++, S-18:1++) degrade to a greater extent (−40% and −90%) than S-14++ (−15%). After 3 months storage at 40 °C, monoesters with fatty acids of C12 or longer decrease, again with longer fatty acids decreasing to a higher extent. Nevertheless, the decreasing rate is a lot higher being around 90% for all fatty acids longer than C14 (Supporting Information Table 2). With an initial value of about 8% for S-12/12++, this diester is the most abundant within the diester fraction (Figure 7C). After storing for 3 months at 40 °C, the S-12/12++ decreases by more than 98% to less than 1% of the total intensity (Figure 7C and Supporting Information Table 2). All other diesters also contribute only approximately 0.1% to the total intensity, losing also more than 90% of the total intensity (Figure 7C and Supporting Information Table 2). The effect of longer fatty acid chains, which degrade to a greater extent, becomes more apparent in samples stored at 25 °C. The enormous effect of high degradation at longer fatty acid chains as well as a higher degree of esterification can also be seen among the triesters (Figure 7D). S-12/12/12++ decreases from about 1.8% initially to about 0.3% after 3 months at 25 °C and to about 0.1% after 3 months at 40 °C storage (Figure 7D and Supporting Information Table 2). In this case, only about 5% of S-12/12/12++ compared to the initial value is still present (Figure 7D). Additionally, for all the other triesters, in general only 20% of the total triester amount compared to the initial value is still present after storage at 25 °C, whereas only 2–6% of the initial value is present after storing at 40 °C (Figure 7D and Supporting Information Table 2). After 3 months at 25 °C, the proportion of triesters decreases by 80%, which is more than the diesters, which only decreased by about 40–70% depending on their fatty acid chain length (Figure 7C,D and Supporting Information Table 2). Interestingly, storage at 40 °C for 3 months does not cause any difference in the degradation rates of monoesters (if longer than C14), di- and triesters anymore.

Overall, triesters degraded noticeably faster than diesters, which in turn degraded faster than monoesters (as already mentioned, there may even be an increase for monoesters with fatty acids shorter than C12). This is mostly evident after storing for 3 months at 25 °C. Comparing monoesters reveals higher degradation rates with increasing fatty acid chain length. Free POE units with and without fatty acids showed the highest increase, especially for POE-00+/POE-08+/POE-10+/POE-12+ (Supporting Information Table 2). Stronger effects were observed after storage at 40 °C, whereas at 25 °C more differences between the fatty acid chain length and degree of esterification were observed.

## Discussion

### Regioselectivity for Polysorbate Degradation—Theory and Experimental Evidence

Oxidative processes are initiated by catalysts such as light or iron, which are present in pharmaceutical production.^80^ In this context, Bensaid et al. (2022) identified metal leachables as a major root cause of oxidative PS degradation.^81^ Both, iron and light have the potential to be involved in the formation of radicals through redox reactions as mentioned above (reaction eqs 1–5 Figure 2), generating reactive oxygen species that ultimately degrade PS20. However, it is important to note that their half-lives vary, with the nonradical ROS (H_2_O_2_ and ROOH) having the longest half-lives, at least at 37 °C, which can be up to 2 h. In contrast, superoxide radicals (^•^O_2_^–^), alkoxy radicals (RO^•^) and singlet oxygen (^1^O_2_) show a half-life of 10^–6^ s at 37 °C. OH^•^ radicals have the shortest half-life of 10^–9^ s at 37 °C, demonstrating their high reactivity leading to rapid interaction/reaction with adjacent molecules.^82,83^ Keeping these reactions in mind helps to understand the degradation pattern of PS20 after analysis by UHPLC-MS and the general discussion of susceptible degradation sites of PS20.

DFT calculations and MD simulations have been carried out to address the issue of oxidation-prone segments within PS molecules. To date, only a few studies have performed MD simulations on PS at all.^84,85^ Nevertheless, the oxidative degradation of polysorbates has not been investigated in these few studies. Within initially performed simulations by sampling the conformational space of individual molecules, no preferred conformation could be identified. Therefore, we analyzed the conformations of PS molecules in terms of their radical susceptibility taken from evenly spaced structures of monomer simulations. Similar results were obtained by Amani et al. (2011), where a simulation of an individual PS80 molecule did not show a predominant conformation.^84^ Therefore, not a single particular conformation, but various diverse conformations of polysorbate, may be equally susceptible to radical degradation of the POE chains.

### Reactivity Prediction

To assess the radical susceptibility of different PS segments, we conducted cDFT calculations. Both the time-resolved (Supporting Information Figure 5) and the convoluted Fukui indexes (Figure 4) of POE carbons show no obvious pattern, only a mere spread slightly above zero. Therefore, the Fukui indices were not able to predict the oxidative degradation of the polysorbate molecules investigated.

### Solvent Accessibility of Polysorbate Segments in Simulations

Another surrogate parameter was chosen, namely the oxidative degradation of PS20 in a micellar environment was studied by examining the accessibility of the surrounding solvent. Afterward, the results were then compared with practical laboratory experiments. Micelles are formed spontaneously above the critical micelle concentration (cmc), with the fatty acid (or acids if there is more than one) forming the hydrophobic core of the micelle with the least contact to the solvent, as shown in Figure 3 (simulation in water). The inaccessibility of the fatty acids to water is reflected in the fact that fatty acid(s) of all simulated systems show the least water contacts during the formation of the interior of the micelle (Figure 5). Similar results have been published in molecular dynamics simulations of PS80 molecules by Amani et al. (2011).^84^ They also observed that once the micelle is formed, it undergoes restructuring to achieve its final assembly structure.^84^ It has been demonstrated that the hydrophobic fatty acid inside the micelle is not always fully stretched.^84^ When the connector chain and the fatty acid are examined together as one chain, the deviation from a straight chain is even more pronounced.^84^ This is a trend we also observed in our simulations. Due to their position within the micelle core, the solvent exposure of fatty acids does not increase when the number of fatty acids is increased (mono- to diester), nor when a more sterically demanding fatty acid is introduced (*lau* vs *ole*). Therefore, based on these data, fatty acids have a lower solvent and therefore ROS accessibility.

### Unesterified Polyoxyethylene (POE) Chains

Also consistent with our simulations, the polar and unesterified POE chains attached to the sorbitan ring are located on the micelle surface, and thus have the most contact with the solvent. Furthermore, these unesterified POE chains were observed to unfold with more freedom compared to the connector POE chain in MD simulations by Amani et al. (2011).^84^ As they have the most contact with water, they are predestined to be easily reached by ROS and are therefore likely to be the most susceptible to oxidative degradation. This would become visible in practical laboratory experiments, as an increased POE-00+ content since POE-00+ reflects POE chains without fatty acid (Supporting Information Table 2). The POE-00+ can be cleaved from the corresponding sorbitan ring to become a free POE chain. However, this conclusion assumes that oxidative cleavage occurs near the sorbitan ring for nonesterified POE chains. In fact, after 3 months at 40 °C, an increase from about 2% initially to 10% of POE-00+ (378% increase) compared to the initial value was observed (Supporting Information Table 2). The assumption that unesterified POE chains have indeed been degraded in their entirety might therefore at first sight appear to be a possible degradation pathway. Nevertheless, the most abundant OE chain length of POE-00+ is initially found to be 12 OE units and decreases to 10 OE units after 3 months of storing at 40 °C. As both unesterified and esterified (connector) POE chains are thought to consist of around 5 OE units, it appears that the unesterified POE chains are shortened over time rather than being completely separated from the PS molecule. Furthermore, such POE chains can be generated by either sorbitan or isosorbide degradation. Additionally, it needs to be mentioned, that free POE chains (POE-00+) can also be formed by hydrolytic cleavage of esterified free POE chains (Figure 1). This study was carried out in Milli-Q water, in the absence of protein, making hydrolytic degradation by host cell proteins unlikely. Chemical hydrolysis is less likely at pH 6, but may not be excluded with decreasing pH values as observed after storing for 3 months at 40 °C (pH decrease from 6.3 to 3.5). This seems likely as POE chains with C18 and C18:1 (POE-18+/18:1+) show decreasing intensities after storing at 25 and 40 °C. Moreover, the increasing rates of POE12+/POE14+/POE16+ are rather low compared to POE-00+, which may be due to further hydrolytic degradation (Supporting Information Table 2). Thus, POE-00+ can originate from both, hydrolytic degradation of esterified free POE chains and oxidative degradation of unesterified POE chains.

Another degradation pathway being discussed for the unesterified POE chains was first described by Donbrow et al. (1978).^27,86,87^ They proposed that the terminal hydroxyl groups of the unesterified POE chains are relatively stable and are only degraded to terminal aldehyde or carboxyl groups when exposed to strongly acidic and strongly oxidizing conditions. Nevertheless, it is reported that hydroperoxide radicals can lead to instability, resulting in short-chain acids and therefore a stepwise degradation of the unesterified POE chain.^27^ Consequently, if a PS molecule initially carries 24 OE units (as detected in our UHPLC-MS measurements), storage at 40 °C for 3 months results in 20 OE units, as observed in our study. This may indicate a loss of OE units at the terminal hydroxyl groups on the unesterified POE chains, resulting in the formation of aldehydes, formic and acetic acid.^86−88^ Indeed, as already mentioned a pH decrease was detected indicating the formation of short chain acids by oxidative cleavage. The formation of small amounts of formic acid through oxidative degradation is a known phenomenon in literature.^33^ Apparently, this mainly affects di- and triester as well as monoester molecules that are esterified to longer fatty acids (from C14 on) (reduction by 4 OE units). Thus, multiple esterification and increasing fatty acid chain length may also be a critical factor in the oxidative degradation of unesterified POE chains. For samples stored at 25 °C, this effect was only observed for monoesters with the unsaturated fatty acid C18:1. There was no apparent loss of the most common OE unit amount, indicating that the ambient conditions (temperature of 25 °C/60% r.h.) were not strong enough to oxidize unesterified POE chains within the examined time frame. Thus, it can be hypothesized that the oxidative degradation of the nonesterified POE chains can occur via two pathways: scission adjacent to the sorbitan ring and oxidation of the terminal hydroxyl groups.

### Connector Polyoxyethylene (POE) Chains

To provide a holistic understanding it must be acknowledged, that the above-discussed oxidative degradation of the unesterified POE chains is not considered to be the most critical point in terms of oxidative degradation. The esterified (or connector) POE chains are stated to be the more critical segment regarding oxidative degradation although they are “pulled” closer to the COM by the very strong tendency of the attached fatty acid to be in the core of the micelle. Thus, they are more shielded from the solvent than the unesterified POE chains (Figures 1 and 3).^89^

Among the systems investigated, the monoester with the shortest fatty acid (*w-lau*) presents the lowest solvent exposure of the connector segment, which increases if replaced by a more sterically demanding one (*w-ole*). Finally, an even greater increase in water contacts is observed when a second fatty acid is introduced in each monomer (*wx-lau*). This is due to the fact that a more sterically demanding fatty acid (lauric acid −12C vs. oleic acid-monounsaturated omega-9 18C), but especially two fatty acids in the core of the micelle, lead to a greater competition for the space in the core of the micelle (Supporting Information Figure 6). In fact, both the *w*- and *x*-POE chains of the diester, show a significantly higher number of contacts with water compared to the *w*-POE chain of the monoesters (Figure 5), suggesting that the connector POE chain may be decisive in explaining the susceptibility to radical attacks in solution. Considering MD simulations of the connector POE chains show that oxidative cleavage is likely to occur in the vicinity of the sorbitan ring, as most water contacts were observed within these units (Figure 6). Additionally, this is further confirmed in practical laboratory experiments as the most abundant OE chain length of the POE-FA+ species (cleaved connector POE chains) decreased from 11 to 12 OE units initially to 5–6 OE units (Supporting Information Figure 4). If the cleavage was adjacent to the fatty acid, the most abundant OE chain length of the POE-FA+ would be 1–2 OE units. Therefore, in contrast to the unesterified POE chains, the putative atoms of oxidative scission within the connector POE chains can be defined with greater certainty. Comparing the number of water contacts of a diester connector with a monoester connector carrying the same fatty acid shows why our experimental results reveal that higher order esters degrade more rapidly. Each connector POE chain is more exposed to solvents when two POE chains are esterified and provides more opportunities for radical attacks. Additionally, as shown in Figure 5, increased water contacts for the sorbitan ring were observed in the presence of diesters in contrast to the monoesters. Thus, the sorbitan ring appears to be more susceptible to oxidative cleavage in this case, suggesting that cleavage of the connector POE chain occurs close to the sorbitan ring and may be more probable for the diesters in general. Similarly, this is again reflected in the water accessibility data of a single connector POE chain, suggesting oxidative cleavage at the OE units adjacent to the sorbitan ring (Figure 6). Moreover, not only are the single connector chains more solvent-exposed in a diester, but there are twice as many as in the corresponding monoester molecule. As a result, the probability of a ROS affecting a connector POE chain is twice as high.

Thus, multiple esterification and longer fatty acids at the respective connector POE chain were shown to lead to increased oxidative degradation. This gets particularly evident in Figure 5, illustrating the higher water accessibility for the longer fatty acid (C18:1). These computational backed assumptions have been confirmed by our practical laboratory experiments. S-12/12++ as well as S-12/14++//S-12/16++//S-12/18++ decreased by ∼70%, whereas the shorter (S-12/08++ and S-12/10++) fatty acids only decreased around 40% (Supporting Information Table 2) after storing for 3 months at 25 °C. Similar results regarding the higher oxidative susceptibility of longer fatty acids have already been reported by several groups.^33,90−92^ They also reported and confirmed that the polyesters (di- and triesters) are primarily affected by oxidative degradation. As longer fatty acids as well as the presence of more fatty acids (di- and triester) contribute to more hydrophobic PS species, they might have a higher tendency to form micelles at lower concentrations. Thus, species with longer fatty acids and species with di- and triesters show lower cmcs as already discussed in literature.^19^ These species might therefore be more susceptible to oxidative degradation, as they exist primarily within micelles rather than as free-floating monomers. Micellar driven oxidation is already discussed in literature especially by Kranz et al. (2020) and Peters et al. (2022).^77,78^ With regard to iron-catalyzed oxidative stress, Kranz et al. (2020) suggested that the reiterating oxygen atoms of the POE chains are an additional decisive factor, as they can form complexes with iron ions that may propagate the oxidative degradation process.^77^ While there is high oxidative degradation within the di- and triester section, the fraction of monoesters increases for S-08/-10/-12++, as the corresponding triesters first turn into diesters and finally into monoesters. Nevertheless, monoesters also decrease when their fatty acid chain length exceeds S-14++, resulting in increasing amounts of the corresponding POE-*XX* chains and S-00++ (Supporting Information Table 2). For the most abundant monoester within PS20 molecules (S-12++) this results in increasing amounts of S-00++ and POE-12+ (205% vs.49% after 3 months at 25 °C). Thus, longer fatty acid chains are not only degraded to a greater extent within the di- and triester fraction, but also within the monoester fraction. This is again consistent with the just mentioned hypothesis of higher oxidative degradation for higher hydrophobic PS species. S-18:1++ shows the highest degradation after 3 months at 25 °C, which is expected due to the unsaturated and long fatty acid. It also shows the highest rate of decreasing within the free POE chains POE-18:1+. This is consistent with our FI calculations, which show that C80/81, corresponding to the carbon atoms of the double bond in oleic acid, have the second highest susceptibility towards radical degradation (Figure 4). The highest monoester degradation of C18:1 (oleic acid) was also recently published by Carle et al. (2024).^90^ Since PS80 contains approximately 5 times more oleic acid than PS20 (Ph.Eur.), it is reasonable to assume that PS80 is more susceptible to oxidation than PS20.^22^ This was also recently demonstrated by Kozuch et al. (2023) in a comprehensive comparative study of PS20 and PS80.^33^

### Presumably Most Crucial Element in Polysorbate Oxidation

Although the unesterified POE chains have greater accessibility to the solvent and are thus assumed to be most affected by oxidative degradation, the effect of cleavage at the connector POE chain is considerably more severe.^93^ The cleavage of such a chain separates the hydrophilic head from the hydrophobic tail of the polysorbate molecule, causing the PS to lose its amphiphilic character. According to the pharmacopoeias any molecule, that is not composed of a sorbitan with a POE unit attached to it and at least one esterified fatty acid, does not meet the criteria for being an intact (amphiphilic) PS molecule.^22,41^ These molecules could be either an impurity or a degradation product and are in our case defined as increasing amounts of POE-FA/00+ and S-00++. In contrast, if an unesterified POE chain is attacked and subsequently shortened by a few OE units, the effect on the amphiphilic character of the PS molecule is expected to be less. As polysorbates protect proteins primarily by virtue of their amphiphilicity, it is initially of minor relevance whether they cover hydrophobic protein patches or, more importantly, occupy hydrophobic surfaces and/or interfaces, thus preventing proteins from particle formation. Once the polysorbate loses its amphiphilicity through oxidative degradation, i.e. when all connector POE chains are cleaved, it is unlikely to be able to maintain its beneficial effect of preventing protein particle formation. The resulting products of this degradation process are the aforementioned S-00++ and the corresponding POE-FA+. Compared to the PS molecule, the S-00++ species are highly hydrophilic due to the absence of the hydrophobic fatty acid chains. They can be further degraded to POE-00+, which are also hydrophilic species. An increase in these species is therefore considered to be particularly serious and relevant as PS may not be able to maintain its protein protective properties. On the other hand, highly hydrophilic, not amphiphilic, species (S-00++, POE-00+) do not form micelles and are therefore assumed to be less susceptible to oxidative degradation. Moreover, as mentioned above, all free POE chains (POE-*XX*+) are not defined as polysorbates but may have surface active properties and form micelles, if they are esterified. Finally, oxidative degradation of nonesterified POE chains and connector POE chains presumably occurs simultaneously.

In this context, PS seems to show its protective behavior when it is esterified at least once (with a connector POE chain) and attached with unesterified POE chains. Thus, degradation of the tri- and diester is undesirable, but may not be that severe as long as monoesters are still present.

## Conclusion

For the first time, a study of the oxidative degradation of polysorbate (PS) has been carried out, comparing and combining both computer simulations and laboratory experiments to gain a comprehensive understanding of PS oxidation on a molecular level that was evaluated in terms of practical relevance. Molecular dynamics (MD) simulations and conceptual density functional theory (cDFT) calculations were performed to assess the oxidative degradation of PS. Within initial simulations no preferential conformation of the PS molecule could be determined. cDFT calculations were not able to reveal differences regarding the radical susceptibility of the different POE chains but demonstrated an elevated propensity for radical attack on the oleic acids’ unsaturated carbons (Figure 5). This finding was confirmed by practical laboratory experiments revealing a higher degradation of C18:1 containing PS species such as S-18:1++ monoesters or degraded POE-18:1+. These longer fatty acid chain species as well as diester molecules and longer fatty acid chains exhibited enhanced solvent accessibility in MD simulations as well as in practical experiments due to a greater competition regarding the space within the micelle core and increasing probability of a radical attack by increasing esterification. Moreover, as they are more hydrophobic species that subsequently show lower cmc values, their existence in form of micelles must be a key factor regarding their oxidative degradation.^19^

Unesterified POE chains were observed to exhibit the highest solvent accessibility in MD simulations compared to esterified (connector) POE chains. Nevertheless, the degradation of a connector POE chain has a considerably greater impact on the performance of the PS regarding protein protection, as the PS molecule may lose its protein protecting properties. Thus, the connector POE chain is stated to be the crucial element of PS oxidation.

The loss of amphiphilicity of the PS molecule results in an increase of S-00++ and POE-FA+, which are not PS species according to pharmacopoeial specifications. Provided that the amphiphilic properties of the surfactant are retained, it is reasonable to assume that PS is able to protect proteins from particle formation even after oxidative degradation of the PS molecule has occurred. Therefore, PS is presumably showing its protective properties regarding protein particle formation if it is at least esterified once (with a connector POE chain) and also contains unesterified POE chains.^94^ This leads to the conclusion that di- and triester degradation is not desired but may not be that severe as long as monoesters are still present.

Apparently, the protein-protective effect of polysorbate is independent of, or in spite of, the considerable heterogeneity of the surfactant. The degradation to more hydrophilic substances, such as S-00++ and POE-00+, suggests that the protective effect of polysorbate is at least partially reduced. Since oxidative degradation also results in amphiphilic degradation products, these might still have protein protective properties. Further studies will be needed to assess the protective effect of these oxidative degradation products of PS.

In general, a more comprehensive understanding of oxidative PS degradation is provided, by combining theoretical and practical results for the first time demonstrating critical oxidation products and the crucial element of PS oxidation.

## Supporting Information Available

SI Figure 1: Simplified scheme of PS degradation fragments. Radical oxidation (red arrows) results in POE or even POE + FA fragments, while pure FA fragments are released through hydrolysis (blue dashed arrows). This scheme is incomplete as it only considers the degradation starting at the *z*-POE unit, subsequently following through the *y*-, *x*-, and *w*-POE units, and degradation within POE units is not considered. SI Figure 2: Segment-wise water contact comparison of diesters with different esterification sites. SI Figure 3: Radius of gyration for every simulated system across the entire simulation. SI Table 1: Composition of polysorbate raw material components/impurities in polysorbate 20 HP according to the European pharmacopoeia (Ph.Eur) 11th edition, and the certificate of analysis for the batch used. SI Table 2: Change of relative intensity compared to initial values for free POE chains, mono-, di- and triester as well as isosorbides after stressing with 10 ppb Fe^2+^ and 100,000 lx·h light and subsequent storing for 3 months at 25 and 40 °C. SI Figure 4: Distribution of OE units within a free POE-12+ chain in a 0.04% PS20 solution in water after initial spiking with 50 ppb Fe^2+^ and exposure to 100,000 lx·h light. Initially a monomodal distribution is observed with the most abundant OE chain length being OE12 (light violet), which changes into a bimodal distribution after storing for 3 months at 25 °C and a monomodal distribution with shorter OE chain length of 5 (dark violet). SI Figure 5: Fukui indexes of the carbons in a PS20 representative molecular structure. SI Figure 6: Radial distribution of the segment’s atoms to the center of mass of the micelle for all simulated systems. The *x*-axis describes the radius in Å, the *y*-axis shows the standard radial distribution. SI Figure 7: The geometry optimized structure of the propionic acid (3,2,2,2) sorbate with the atomic labels. SI Figure 8: Radical susceptibility *f*^0^ for the conformer ensemble in implicit water and ether (PDF)

## Abbreviations

(c)DFT: (conceptual) density functional theory
API: active pharmaceutical ingredient
B3LYP: Becke, 3-parameter, Lee–Yang–Parr
C12:0: lauric acid
C18:0: stearic acid
C18:1: oleic acid
cmc: critical micelle concentration
CMR: critical micelle concentration range
ESI: electron spray ionization
FA: fatty acid
FI: Fukui index
FMO: frontier molecular orbital
HP: high purity
MD: molecular dynamics
MS: mass spectrometry
*NpT*: isothermal–isobaric ensemble
*NVT*: canonical ensemble
OE: oxyethylene
POE: polyoxyethylene
POE-00+: single POE chain without fatty acid, single charged
POE-12+: single POE chain with C12 (lauric acid), single charged
PS: polysorbate
PS20: polysorbate 20
PS80: polysorbate 80
QDa: quadrupole Dalton based mass detector
r.h.: relative humidity
ROS: reactive oxygen species
RP-UHPLC-MS: reversed-phase ultra high-performance liquid chromatography system coupled with a quadrupole Dalton based mass detector
S-00++: sorbitan without fatty acid, but OE units, double charged
S-12++: sorbitan with C12 (lauric acid) and OE units, double charged
SASA: solvent accessible surface area
SPME: smooth particle-mesh Ewald
TIP4P-EW: transferable intermolecular potential 4 points-extended water
*w*-lau: polysorbate molecule(s) with exclusively lauric acid esterified to the *w*-POE chain
*w*-ole: polysorbate molecule(s) with exclusively oleic acid esterified to the *w*-POE chain
*wx*-lau: polysorbate molecule(s) with exclusively lauric acid esterified to *w*- and *x*-POE chains
xTB: extended tight-binding
