# Supplementary material for: Micellar Solvent Accessibility of Esterified Polyoxyethylene Chains as Crucial Element of Polysorbate Oxidation: A Density Functional Theory, Molecular Dynamics Simulation and Liquid Chromatography/Mass Spectrometry Investigation
Source: Mol Pharm. 2025 Feb 3;22(3):1348–64. doi: 10.1021/acs.molpharmaceut.4c01015 (PMC11881146; doi:10.1021/acs.molpharmaceut.4c01015)
Supplement: Supplementary file 1 — mp4c01015_si_001.pdf [file mp4c01015_si_001.pdf]

Supplementary Information

Micellar Solvent Accessibility of Esterified Polyoxyethylene Chains as Crucial Element of Polysorbate Oxidation: A Density Functional Theory, Molecular Dynamics Simulation and Liquid Chromatography / Mass Spectrometry Investigation

Johanna Weber[⊥, 1], Leonardo Pedri[⊥, 2], Luis P. Peters[2], Patrick K. Quoika[3], Dennis F. Dinu[2], Klaus R. Liedl[*, 2], Christofer S. Tautermann[4], Tim Diederichs[5] and Patrick Garidel[*, 5]

[1]Institute of Pharmacy, Faculty of Biosciences, Martin-Luther-University Halle-Wittenberg, 06120 Halle, Germany

[2]Department of General, Inorganic and Theoretical Chemistry, University of Innsbruck, 6020 Innsbruck, Austria

[3]Center for Protein Assemblies (CPA), Physics Department, Chair of Theoretical Biophysics, Technical University of Munich, 85748 Garching, Germany

[4]Boehringer Ingelheim Pharma GmbH & Co. KG, Medicinal Chemistry, 88400, Biberach/Riss, Germany

[5]Boehringer Ingelheim Pharma GmbH & Co. KG, Innovation Unit, PDB-TIP, 88400, Biberach/Riss, Germany

[⊥]shared first authorship

[*]corresponding authors

Klaus.Liedl@uibk.ac.at

and

Patrick.Garidel@boehringer-ingelheim.com

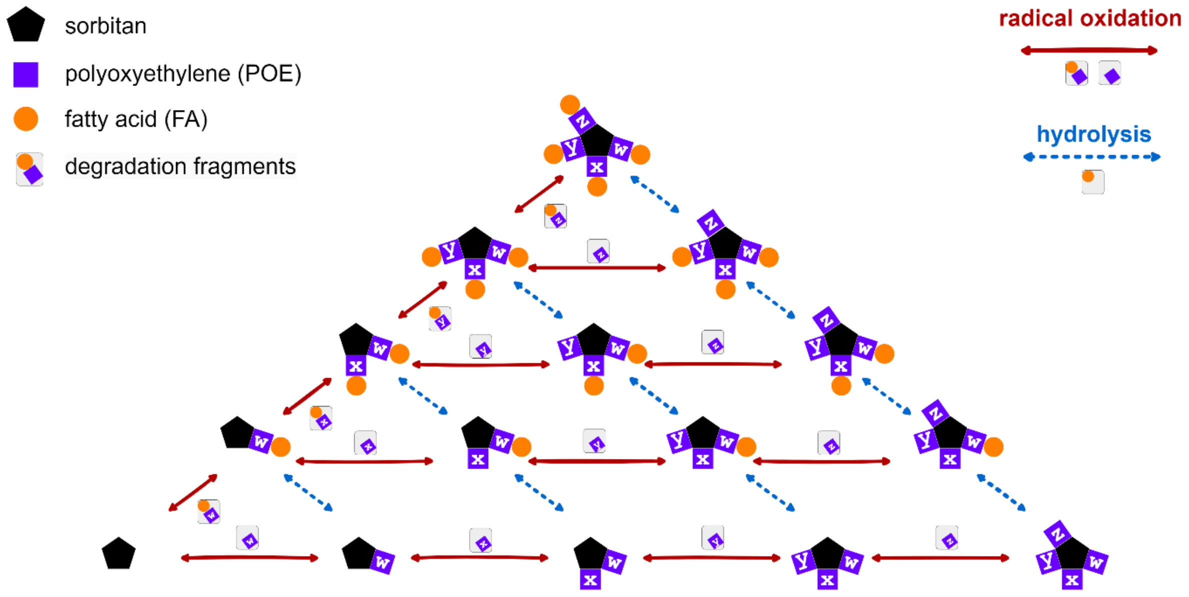

SI Figure 1: Simplified scheme of PS degradation fragments. Radical oxidation (red arrows) results in POE or even POE+FA fragments, while pure FA fragments are released through hydrolysis (blue dashed arrows). This scheme is incomplete as it only considers the degradation starting at the z-POE unit, subsequently following through the y-, x-, and w-POE units, and degradation within POE units is not considered.

Water contacts comparison between *wx/wy/wz*-diesters

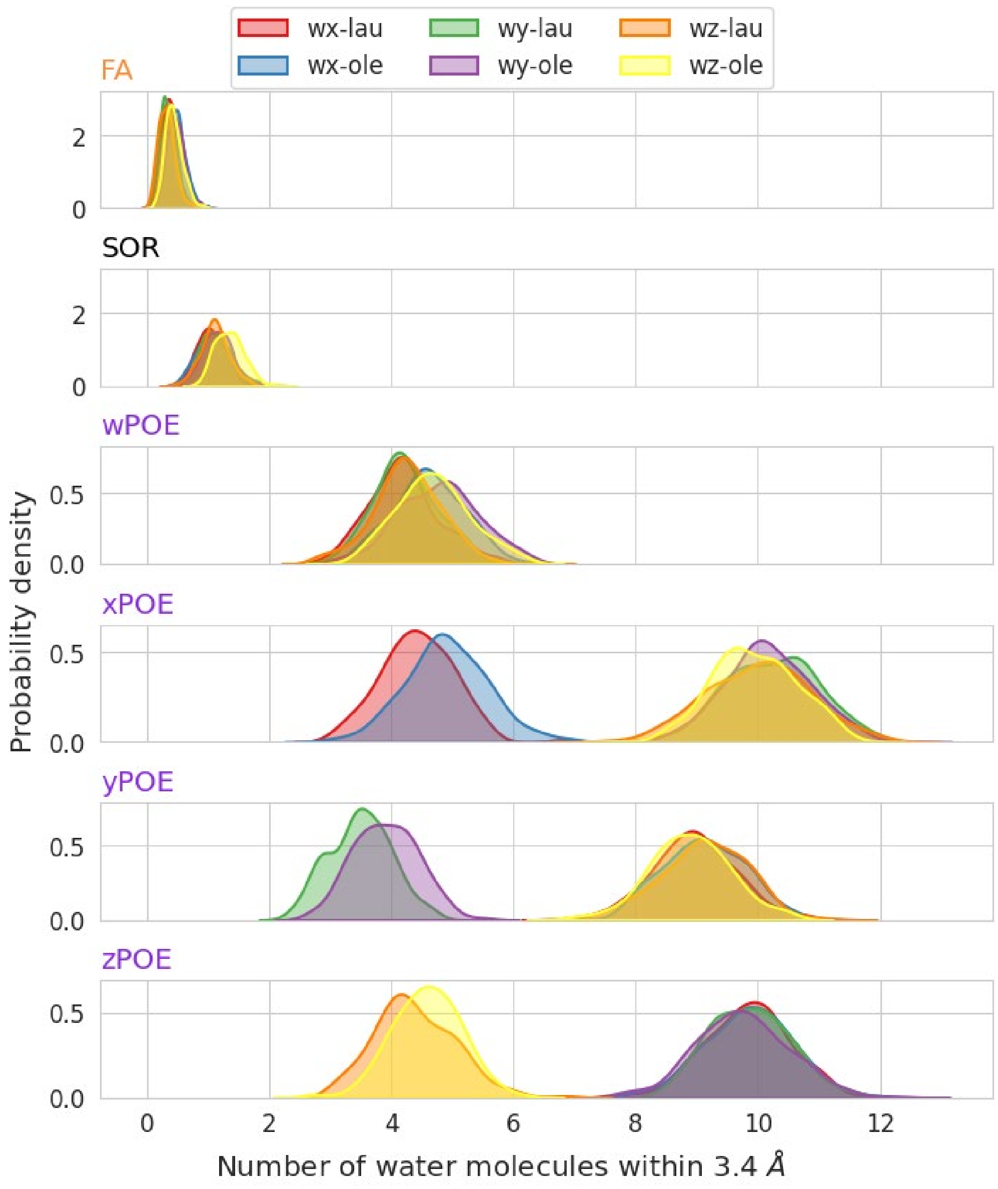

SI Figure 2: Segment-wise water contact comparison of diesters with different esterification sites.

The impact of the position of the second esterification was also investigated by comparing diester systems (*wx-, wy-, wz-lau* & *wx-, wy-, wz-ole*) amongst each other (**SI Figure 2**). The comparison in **SI Figure 2** shows how the esterification site's role appears limited to the changing esterified POE chain. This can be seen by comparing the diesters with the same fatty acid amongst each other, where it becomes apparent,

that only the distribution of the *x/y/z*-POE chain where one system possesses a fatty acid attached changes, and the others do not. Comparing the laurate (lau) diesters with the oleate (ole) diesters, i.e., diesters with different fatty acids, the same conclusions can be drawn as in the comparison of the laurate monoester with the oleate monoester (monoesters with different fatty acids). The water contacts distributions of the POE connector chains of the mono-/di-ester with the smaller fatty acid are always shifted to lower values, meaning less contact with the solvent.

Adaptation of Maestro generated Desmond (.cms) files to GROMACS (.gro) files

After converting the Maestro generated Desmond forcefield file (.cms) to GROMACS (.gro) using ‘intermol convert’, the following steps must be taken:

1. Copy the oplsaa.ff directory provided by GROMACS into your working directory
2. Copy the converted *.top file and rename it to *.itp
3. Move [atomtypes] section from *.itp to oplsaa.ff/ffnonbonded.itp
4. Prepare oplsaa.ff/atomtypes.atp
5. Move [system] and [molecules] to from *.itp to *.top
6. Adapt the number of molecules in the *.top file.
7. Add the #include directives in the *.top file to link the required .itp force-field files.

Simulation Protocol

First, 50’000 steps of steepest descent energy minimization were performed. If a maximum force lower than 1’000 $kJ\cdot mol^{-1}\cdot nm^{-1}$ was reached, the minimization was terminated before the completion of all steps. A 500 ps temperature equilibration at 300 K in the canonical ensemble (NVT) followed. It was carried out with the velocity rescaling algorithm and a time constant for temperature coupling of 0.1 ps.[4] Next, the pressure was also equilibrated with a 500 ps equilibration at 1 bar in the isothermal-isobaric (NpT) ensemble. The pressure was controlled with the Parinello-Rahman barostat in an isotropic fashion with a time constant for pressure coupling of 2.0 ps.[5] After both equilibrations, a production run of 1 µs in length was carried out.

Micelle simulation initial molecule arrangement

When building the 3x3x3 cube of monomers to simulate micelle self-assembly, the diameter of the conformation with the largest bounding box of the 27 molecules was determined. Then it was used to set the spacing between each molecule, which was set to be the diameter of the largest conformation with an additional angstrom of space, to prevent possible clashes.

Detailed description of the water contacts calculation procedure

The segment-wise water contacts were calculated by counting the number of water molecules within 3.4 angstrom of the segment's atoms (distances calculated only for "heavy" atoms, i.e., not hydrogen). The 3.4 angstrom cutoff was chosen to include a typical hydrogen bond length with an additional small margin. For every frame of the simulation, the water contacts of all polysorbate molecules were calculated, summed up, and finally divided by the number of polysorbate molecules to yield an average. To correct the autocorrelation, the minimum lag time to de-correlate the data was determined and the contacts timeseries filtered accordingly. Finally, the two-sample Kolmogorov-Smirnov test with two-sided null hypothesis and $\alpha = 0.001$ was used to determine whether the decorrelated water contacts distribution of the same segment for various systems was statistically equal or different.

The atom-wise water contacts of the connector POE chain (i.e., POE chain with attached fatty acid) were calculated in the same fashion as the segment-wise contacts. Instead of calculating the contacts for all the segment's atoms simultaneously, they were calculated for each individual atom in the connector chain.

Radius of gyration of the entire simulation

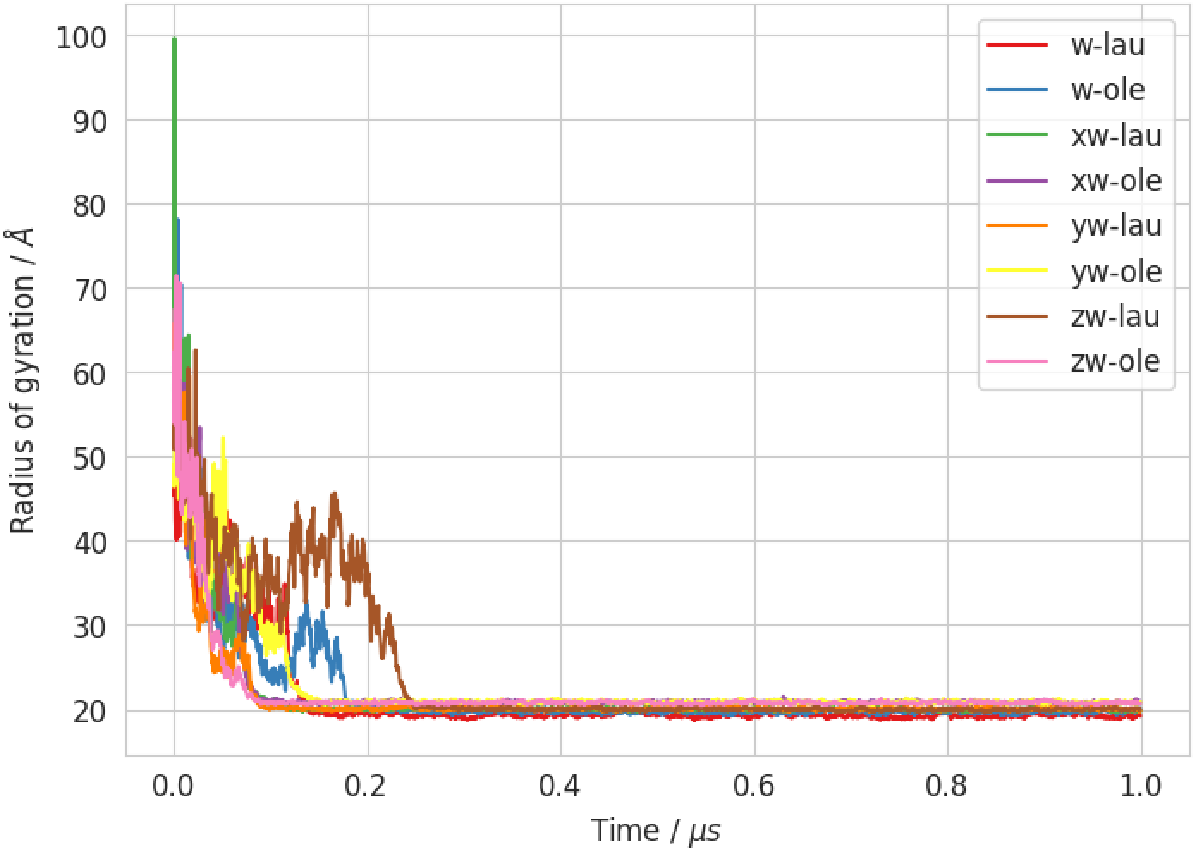

SI Figure 3: Timeseries of the radius of gyration for every simulated system across the entire simulation. It can be used to determine the moment of micelle formation. For the *zw-lau* system, as the micelle is not formed yet after 200 ns, the first 250 ns of simulation time were cut and just 750 ns were used for analysis (compared to the 800 ns of every other system).

SI Figure 3 shows the timespan of the micelle self-assembly process observed in the molecular dynamics simulations. The initial part, typically before 200 ns, presents a very high radius of gyration, meaning that the micelle is not yet formed. Once self-assembly has completed, all polysorbate molecules are found to be part of the micelle and maintain that conformation for the remaining simulation.

	PS20 HP	
	Ph. Eur	Certificate of Analysis CoA
**Acid value / mg KOH·g^{-1}**	≤ 2.0	1.1
**Saponification value / mg KOH·g^{-1}**	40-50	46
**Hydroxyl value / mg KOH·g^{-1}**	96-108	101
**Peroxide value / meqO_2·kg^{-1}**	≤ 10.0	0.0
**Caproic / %**	≤ 1.0	< 0.1
**Caprylic / %**	≤ 10.0	2.7
**Capric / %**	≤ 10.0	3.1
**Lauric / %**	40.0-60.0	54.1
**Myristic / %**	14.0-25.0	17.9
**Palmitic / %**	7.0-15.0	11.3
**Stearic / %**	≤ 7.0	5.0
**Oleic / %**	≤ 11.0	4.9
**Linoleic / %**	≤ 3.0	< 0.1
**Ethylene oxide / ppm**	≤ 1	≤ 1
**Dioxane / ppm**	≤ 10	≤ 1
**Heavy metal / ppm**	≤ 10	≤ 10
**Water / %**	≤ 3.0	0.2

SI Table 1: Composition of polysorbate raw material components/impurities in polysorbate 20 HP according to the European pharmacopoeia (Ph. Eur) 11th edition, and the certificate of analysis (CoA) for the batch used.

Free POE units	After storing for 3 m at 25°C / %	After storing for 3 m at 40°C / %
**POE-00+**	+68	+378
**POE-08+**	+64	+264
**POE-10+**	+427	+193
**POE-12+**	+49	+71
**POE-14+**	+42	+43
**POE-16+**	-7	+57
**POE-18+**	-9	-36
**POE-18:1+**	- 78	--82
**PS20 Monoester**	**After storing for 3 m at 25°C / %**	**After storing for 3 m at 40°C / %**
**S-00+**	+205	+595
**S-08+**	+81	+101
**S-10+**	+60	+60
**S-12+**	+29	-18
**S-14+**	-15	-88
**S-16+**	-38	-92
**S-18+**	-7	-95
**S-18:1+**	-90	-94
**PS20 Diester**	**After storing for 3 m at 25°C / %**	**After storing for 3 m at 40°C / %**
**S-12/08++**	-42	-92
**S-12/10++**	-40	-97
**S-12/12++**	-71	-99
**S-12/14++**	-72	-99
**S-12/16++**	-74	-99
**S-12/18++**	-68	-96

PS20<br>Triester	After storing for 3 m at 25°C / %	After storing for 3 m at 40°C / %
S-12/12/12++	-83	-94
S-12/12/14++	-81	-98
S-12/12/16++	-79	-97
**PS20<br>Isosorbides**	**After storing for 3 m at 25°C / %**	**After storing for 3 m at 40°C / %**
I00+	+57	253
I08+	+31	62
I10+	+30	32
I12+	0	-55
I14+	-33	-89
I16+	16	-92
I18+	24	-93
I18:1+	-31	-97
I12/12+	-64	-96
I12/14+	-72	-97
I12/16+	-60	-92

SI Table 2: Change of relative intensity compared to initial values for free POE chains, Mono- , Di- and Triester after stressing with 10 ppb Fe^{2+} and 100’000 lx·h light and subsequent storing for 3 months at 25 °C and 40 °C.

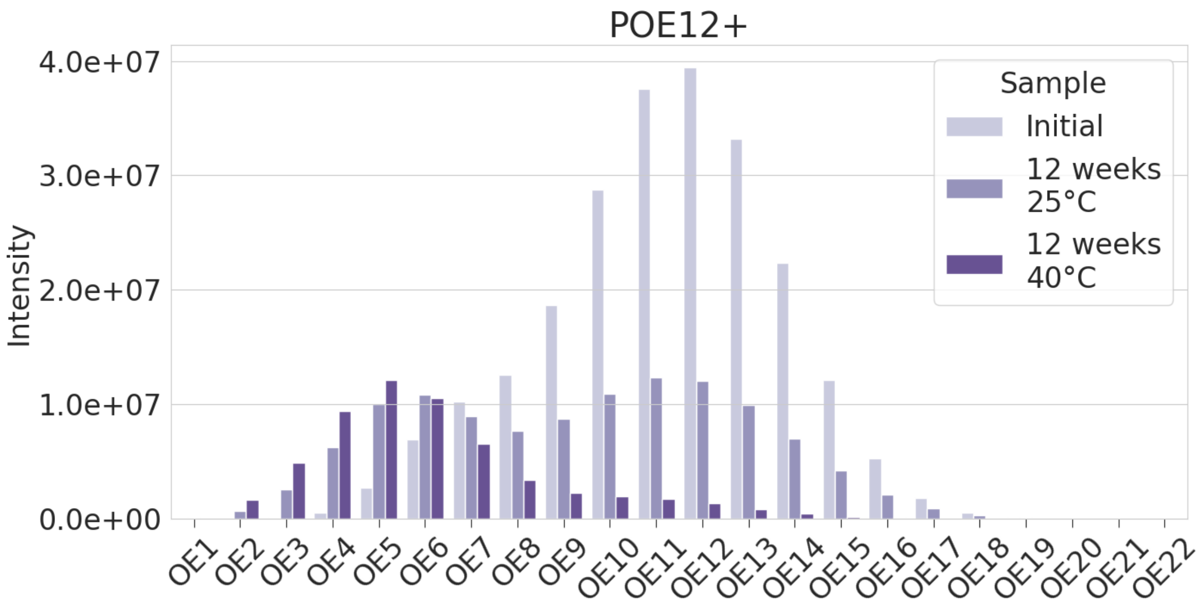

SI Figure 4: Distribution of OE units within a free POE-12+ chain in a 0.04 % PS20 solution in water after initial spiking with 50 ppb Fe^{2+} and exposure to 100'000 lx·h light. Initially a monomodal distribution is observed with the most abundant OE chain length being OE12 (light violet), which changes into a bimodal distribution after storing for 3 months at 25°C and a monomodal distribution with shorter OE chain length of 5 (dark violet).

SI Figure 4 shows the OE units within a free POE chain (being a degradation product or initial impurity) that are Gaussian distributed, initially and after 3 months of storing at 40 °C monomodally. After 3 months of storage at 25 °C, this Gaussian distribution changes into a bimodal distribution for all POE chains with a fatty acid that is longer than C10. In this case, two most abundant OE chain lengths occur, mostly at OE11 and OE5. After 3 months of storage at 40 °C, however, a shift to shorter OE chains is detected, that is resulting in a monomodal distribution with the most abundant OE chain length being 5. It can be recognized from an OE chain length of OE-08 on with shifts to even shorter OE chain lengths with increasing fatty acid length. The most common OE chain length is then no longer 11-12 OE units, but 5-6 OE units (3 OE

units at POE-18:1+). On the one hand, the number of OE units of the free POE-XX+ chains may decrease, which may occur with radicals being present as described by Donbrow et al.(1978). [1] This would result in POE-XX chains with less than 11-12 OE units. On the other hand, this also demonstrates how the degraded connector POE chains, which carry approx. 5-6 OE units, are reflected in form of degradation products. Even if longer fatty acids have a comparatively lower CMC due to the hydrophobic effect and are therefore more likely to form micelles than their short-chain equivalents, it is still unclear whether this micelle formation leads to increased oxidative degradation.[2,3] Based on this data, the limitations of the conducted practical experiments are shown. Even if the degradation products were clearly identified, several explanations exist on how these degradation products are formed. In particular, the exact site of oxidative cleavage of non-esterified POE chains remains uncertain despite simultaneous conducted MD simulations.

Additional information on cDFT calculations

To identify conformations of PS20 more prone to a radical attack, the Fukui index was calculated for every 50th frame of the cMD of PS20 as a monomer. The wavefunction was generated employing tight-binding calculations (xTB), the atomic partial charges evaluated with the Loewdin population analysis in MultiWFN. The resulting Fukui indexes are shown in **SI Figure 5**.

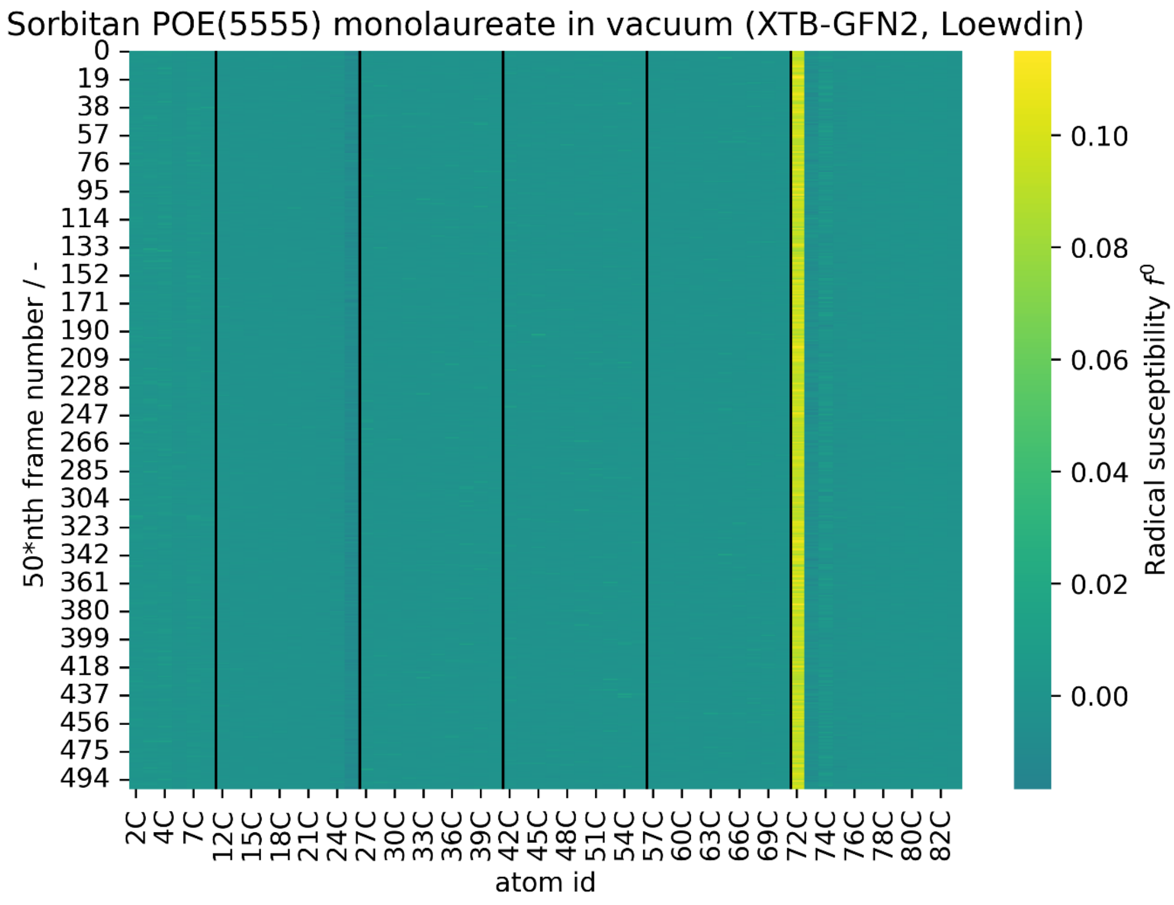

SI Figure 5: The Fukui Indexes of the carbons in a PS20 representative molecular are shown. Starting on the left are the sorbitan carbon, followed by the respective *w, y, x*, and *z* polyoxyethylene carbons and finally the lauric acid carbons - separated with black lines for visual clarity.

Additional to the classical MD simulations, the conformational space of a representative polysorbate with the configuration 3,2,2,2 (*w,x,y,z*) esterified with

propionic acid in the xtb driver package CREST was sampled using the GFN force field variant. CREST was run twice with the default settings, each one time with the implicit solvent models GBSA(water) and GBSA(ether). An extended run time of 800 picoseconds was used, as CREST is limited by default to 500 ps meta-dynamic simulations.

Radial distribution function of polysorbate segments to the center of mass of the micelle

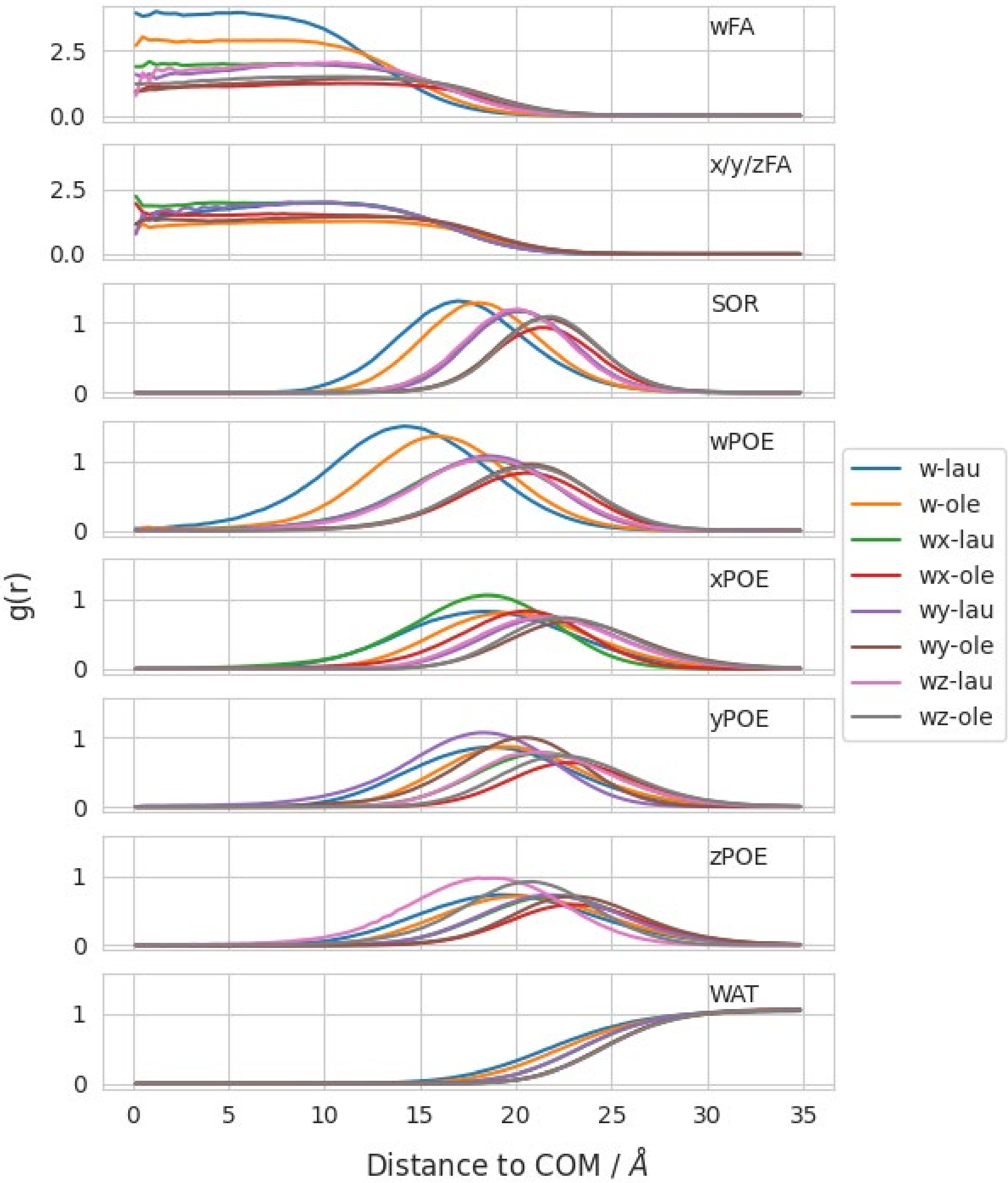

SI Figure 6: Radial distribution of the segment's atoms to the center of mass (COM) of the micelle for all simulated systems. The x-axis describes the radius in Å, the y-axis shows the standard radial distribution.

The radial distribution analysis and segment-wise water contacts analyses shown in **SI Figure 2** were also performed for *wx-, wy-, wz*-di-lau and *wx-, wy-, wz*-di-ole systems. Since the difference between a shorter fatty acid (laurate) and a more sterically demanding one (oleate) can already be seen comparing monoesters amongst each other (*w*-lau & *w*-ole) the comparison of diester systems (*wx-, wy-, wz-lau* & *wx-, wy-, wz-ole*) was omitted within the paper, as it displays the same trend just for two fatty acids being esterified (**SI Figure 2 & 6**).

SI Figure 6 shows how the fatty acids, no matter if short (laurate) or longer (oleate), mono- or diester, always make up the core of the micelle and are thus sheltered from the water. Moreover, the *w*-POE chain, a connector in all systems shown in **SI Figure 6**, is noticeably closer to the center of mass (COM) of the micelle for the monoester systems, further away for the *wx/wy*-diesters and furthest away for the *wz*-diesters. The same trend is displayed by the sorbitan segment. For the *x/y/z*-POE chains, the monoester distributions are again closer to the COM due to the smaller core compared to a diester micelle. For the diesters, the esterified *x/y/z*-POE chain distribution is shifted towards smaller radii, i.e. closer to the core of the micelle compared to a free POE chain, with the trend being more pronounced for the laurate diester compared to the oleate diester. The free POE chains show a less systematic arrangement and are very similar amongst all diesters.

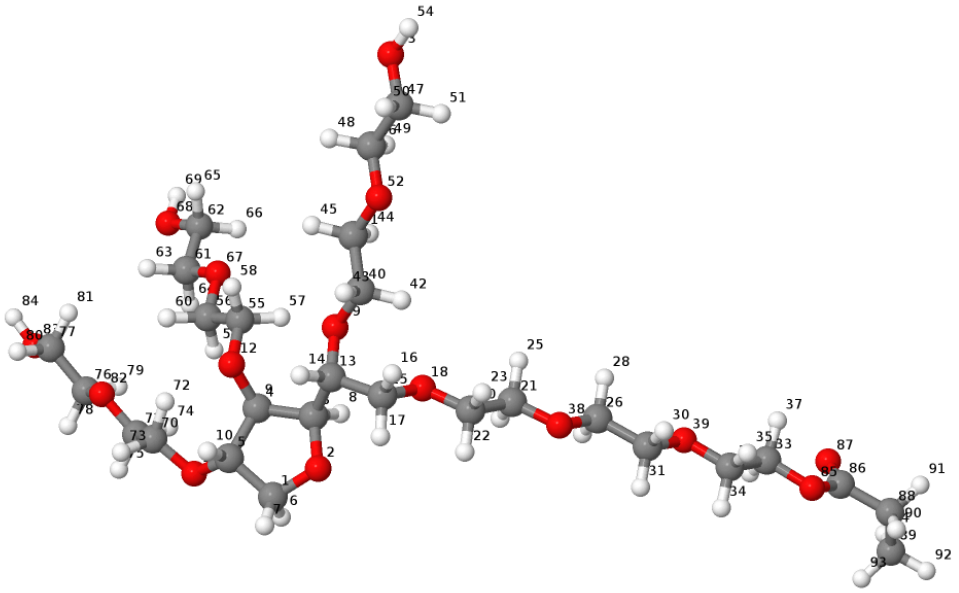

SI Figure 7: The geometry optimized structure of the propionic acid (3,2,2,2) sorbate with the atomic labels.

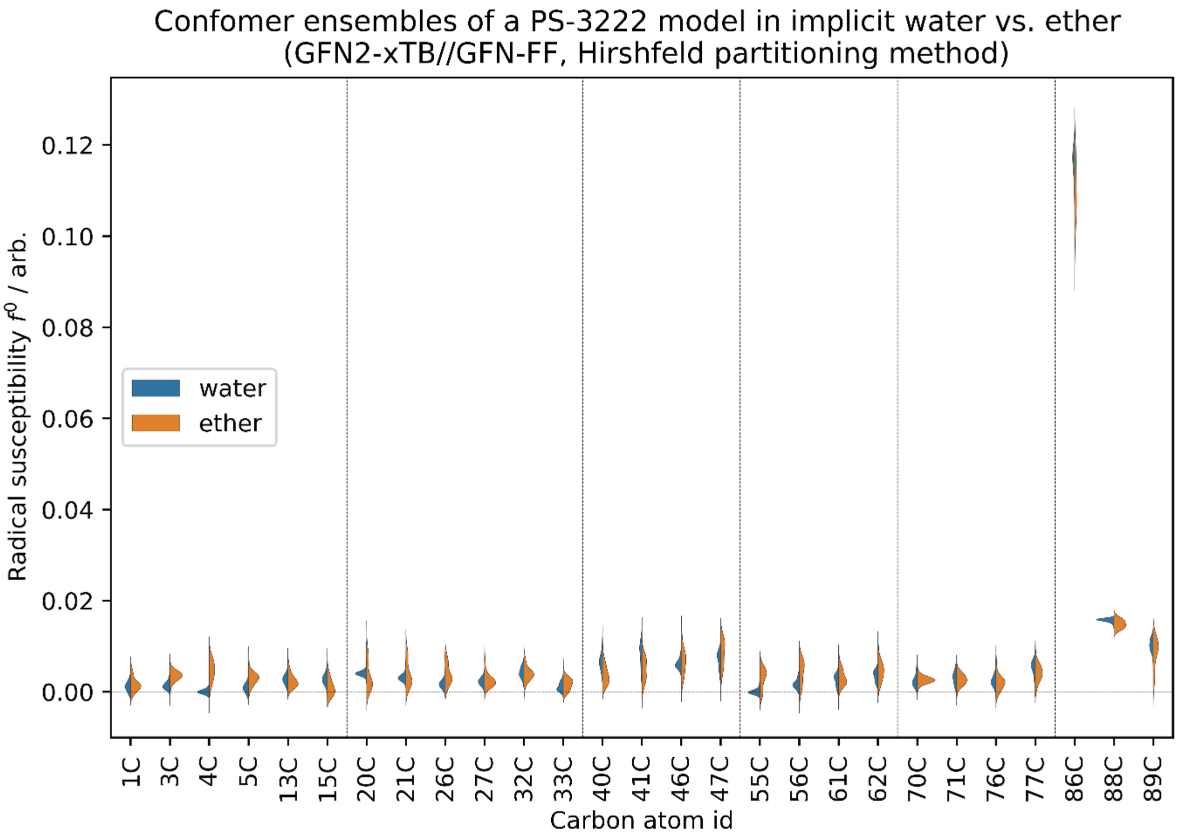

SI Figure 8: The radical susceptibility f^0 for the conformer ensemble in implicit water and ether. The water conformers ensemble f^0 is shown on the left-hand violins in blue and the ethers on the right-hand in orange.

cDFT was shown that it retrieves a plethora of chemical concepts deeply embedded in the chemical understanding such as softness, hardness, electrophilicity, nucleophilicity and also in our case a way to describe the likelihood of a certain site in a molecule to be oxidized by radical stress.[6–8] The reactivity in terms of electro- and nucleophilicity can be calculated either as global or local quantities. Fukui indexes (FI) were initially intended to evaluate the reactivity based on a relaxed structure, which represents in best case the global minimum. The Fukui function f^0 in its condensed form was used to quantify the local response to a radical attack. The Fukui indexes (FI) investigated, depend on the most nucleophile and electrophile sites within a

molecule, as they are the mean of f^+ and f^-. Hence f^0 is also governed by the nucleo- and electrophilicity of the molecule. In general carbonyl carbons are known to be prone to nucleophilic attacks and the electron-rich double bond to electrophilic attacks, which is illustrated in higher FI values shown in **Figure 4**.[9]

$$f_k^+ = |q_k(n+1) - q_k(n)|$$

$$f_k^- = |q_k(n) - q_k(n-1)|$$

$$f_k^0 = -\frac{1}{2}(f^+ - f^-)$$

Calculating Fukui indexes can be conducted with different theory levels and basis sets, predominantly standard DFT such as B3LYP and a 6-311G(d,p) gaussian type orbital basis set for small pharmaceuticals (leads) or for bigger biologics semi-empirical methods, such as extended tight-binding (xTB) with or without implicit solvent. Generally, reactivity descriptors such as the Fukui function depend on the conformation. In the case of flexible molecules, efficient calculation methods such as the tight-binding method are necessary, and also applicable to yield a sufficient sample size of the conformational space. Sampling the conformational space of the PS molecules resulted in monomodally distributed functions.

Different subsequent methods to account the electron density atom-wise are, e.g., Mulliken, Loewdin, and more sophisticated NBO, and Hirshfeld partition method. Different methods were tested and GFN2-xTB with the Hirshfeld partitioning method was chosen to calculate the condensed Fukui function as a good compromise between cost and accuracy.

The Fukui indexes of PS20 are spread broader and the PS80 values are slightly smaller due to the nature of the Fukui index calculation since the density of a single electron is distributed over all atoms with PS80 consisting of more atoms.

References

1. Donbrow M, Azaz E, Pillersdorf A. Autoxidation of polysorbates. *J Pharm Sci*. 1978;67(12):1676-1681. doi:10.1002/jps.2600671211

2. Ferrer M, Comelles F, Plou FJ, et al. Comparative surface activities of Di- and trisaccharide fatty acid esters. *Langmuir*. 2002;18(3):667-673. doi:10.1021/la010727g

3. Stillwell W. Membrane Polar Lipids. *An Introd to Biol Membr*. Published online 2016:63-87. doi:10.1016/b978-0-444-63772-7.00005-1

4. Bussi G, Donadio D, Parrinello M. Canonical sampling through velocity rescaling. *J Chem Phys*. 2007;126(1):014101. doi:10.1063/1.2408420

5. Parrinello M, Rahman A. Polymorphic transitions in single crystals: A new molecular dynamics method. *J Appl Phys*. 1981;52(12):7182-7190. doi:10.1063/1.328693

6. Bendjeddou A, Abbaz T, Gouasmia A, Villemin D. Molecular Structure, HOMO-LUMO, MEP and Fukui Function Analysis of Some TTF-donor Substituted Molecules Using DFT (B3LYP) Calculations. *Int Res J Pure Appl Chem*. 2016;12(1):1-9. doi:10.9734/irjpac/2016/27066

7. Koné MG richard, Bédé AL, Ouattara B, Stéphane G, Karamanis P, Ziao N. Studies of the Chemical Reactivity of a Series of Benzimidazolyl - Chalcone by Quantum Chemical Approaches. *J Drug Deliv Ther*. 2023;13(10):46-53.

8. Roy I, Patel A, Kumar V, et al. Polysorbate degradation and particle formation in a high concentration mAb: Formulation strategies to minimize effect of enzymatic polysorbate degradation. *J Pharm Sci*. 2021;110(9):3313-3323. doi:10.1016/j.xphs.2021.05.012

9. Taylor P. Nucleophilic attack at the carbonyl group. In: *Mechanism and Synthesis*. ; 2003:21-55. https://doi.org/10.1039/9781847557858-00021
